# Predictive Models for the Binary Diffusion Coefficient at Infinite Dilution in Polar and Nonpolar Fluids

**DOI:** 10.3390/ma14030542

**Published:** 2021-01-23

**Authors:** José P. S. Aniceto, Bruno Zêzere, Carlos M. Silva

**Affiliations:** CICECO—Aveiro Institute of Materials, Department of Chemistry, University of Aveiro, 3810-193 Aveiro, Portugal; joseaniceto@ua.pt (J.P.S.A.); brunozezere@ua.pt (B.Z.)

**Keywords:** diffusion coefficient, machine learning, modeling, nonpolar, polar, prediction

## Abstract

Experimental diffusivities are scarcely available, though their knowledge is essential to model rate-controlled processes. In this work various machine learning models to estimate diffusivities in polar and nonpolar solvents (except water and supercritical CO_2_) were developed. Such models were trained on a database of 90 polar systems (1431 points) and 154 nonpolar systems (1129 points) with data on 20 properties. Five machine learning algorithms were evaluated: multilinear regression, *k*-nearest neighbors, decision tree, and two ensemble methods (random forest and gradient boosted). For both polar and nonpolar data, the best results were found using the gradient boosted algorithm. The model for polar systems contains 6 variables/parameters (temperature, solvent viscosity, solute molar mass, solute critical pressure, solvent molar mass, and solvent Lennard-Jones energy constant) and showed an average deviation (AARD) of 5.07%. The nonpolar model requires five variables/parameters (the same of polar systems except the Lennard-Jones constant) and presents AARD = 5.86%. These results were compared with four classic models, including the 2-parameter correlation of Magalhães et al. (AARD = 5.19/6.19% for polar/nonpolar) and the predictive Wilke-Chang equation (AARD = 40.92/29.19%). Nonetheless Magalhães et al. requires two parameters per system that must be previously fitted to data. The developed models are coded and provided as command line program.

## 1. Introduction

Diffusivities are important properties for the proper design, simulation and scale-up of rate-controlled separations and chemical reactions, where they are required for the estimation of dispersion coefficients, convective mass transfer coefficients, and catalysts effectiveness factors [1,2,3]. However, diffusivity data is scarce both in terms of compounds and operating conditions, leading to the need of accurate models capable of predicting diffusivities when no experimental data is available [4].

Currently the Wilke-Chang model [5], proposed in 1955, remains the most widely used equation to estimate binary diffusivities mainly due to its simplicity. It requires only knowledge of solvent viscosity, solute molar mass, solute volume at normal boiling point and operating conditions like temperature. Other hydrodynamic equations have been proposed such as Scheibel [6], Tyn-Calus [7], and Hayduk and Minhas [8]. Correlative models validated for both polar and nonpolar systems have been put forward by Magalhães et al. [9,10], and one may also cite the 2-parameter correlation of Dymond–Hildebrand–Batschinski (DHB) [11,12], based on the free-volume theory, for nonpolar and weakly polar systems at moderate densities. However, these correlations require that data of a given system is available in order to interpolate and extrapolate diffusivities for the desired condition. Hybrid models are also available, such as the predictive Zhu et al. [13] and the predictive Tracer Liu-Silva-Macedo (TLSM) and its 1-parameter correlations (TLSM_d_ and TLSM_en_) [4,14,15]. These are Lennard-Jones fluid models and comprehend two contributions: a free-volume part and an energy component.

With the increase of readily available computing power, Artificial Intelligence and machine learning (ML) techniques have been increasingly applied for the estimation of physical properties of various compounds. In the chemistry field, machine learning is commonly applied in the scope of quantitative structure-property relationship (QSPR) or quantitative structure-activity relationship (QSAR) studies. These are regression or classification models that relate the structure and physicochemical properties of a molecule with a desired response: a chemical property, in the case of QSPR, or a biological activity, in the case of QSAR. QSPR/QSAR approaches have been applied to predict the diffusivity of pure chemicals [16] and acids in water [17] using a database of 320 chemicals and 65 acids, respectively. In both cases, a genetic algorithm was employed to select the molecular descriptors while feed-forward and radial basis function neural networks were used to build the diffusion coefficients models. A squared correlation coefficient above 0.98 was obtained for the test set in either case. Beigzadeh and coworkers [18] developed a feed-forward artificial neural network to estimate the Fick diffusion coefficient in binary liquid systems, using a database of 851 points. Results showed superior performance when compared with other theoretical and empirical correlative models commonly used, with a total average relative deviation of 4.75%. Eslamloueyan and Khademi [19] used a database of 336 experimental data points to developed a feed forward neural network to predict binary diffusivities of gaseous mixtures at atmospheric pressure as a function of temperature and based on the critical temperature, critical volume and molecular weight of each component. This model showed a relative error of 4.47%, lower than other alternative correlations. A QSRP model by Abbasi and Eslamloueyan [20] applied a multi-layer perceptron (MLP) neural network and an adaptive neuro-fuzzy inference system (ANFIS) to estimate the binary diffusion coefficients of liquid hydrocarbons mixtures. These models were constructed on a database of 345 experimental points and showed very good accuracies, with average absolute relative deviation (AARD) of 7.79% for the test data, when compared with five semi-empirical correlations, such as the Tyn-Calus and Wilke-Chang equations. Another QSPR model with five parameters based on genetic function approximation has been proposed to predict diffusion coefficient of non-electrolyte organic compounds in air at ambient temperature [21]. It used a dataset of 4579 organic compounds and provided a very low AARD of 0.3%. The authors applied leverage value statistics to define the applicability domain of the final model. A neural network model based on mega-trend diffusion algorithm was proposed to predict CO_2_ diffusivity in biodegradable polymers [22]. It showed better precision when compared with free-volume and conventional back-propagation models. More recently, machine learning and neural networks models have also been applied to the estimation of the thermal diffusivity of hydrocarbons [23], aromatic compounds insulating material [24], and diffusivity of solutes in supercritical carbon dioxide [25].

In this work we develop models for the prediction of binary diffusion coefficients in polar and nonpolar systems by employing several machine learning algorithms, such as decision tree, nearest neighbors and ensemble methods. A large database of experimental data was collected, divided into polar and nonpolar systems, and used for training the models. The database comprehends experimental points for liquids (except water), compressed gases and supercritical fluids (except CO_2_). Water was excluded due to its usual distinct behavior from other polar solvents, and the large amount of experimental data available for aqueous systems may cause a bias in the model. This later argument also applies to binary diffusivities in supercritical CO_2_. Results were compared with four methods to estimate diffusivities: two hydrodynamic equations (Wilke-Chang and Tyn-Calus), a 2-parameter correlation (Magalhães et al.), and a hybrid model (Zhu et al.).

## 2. Theory and Methods

The methodology used in this work to develop machine learning (ML) models for the prediction of diffusivities can be summarized in the following steps: (i) variable selection; (ii) learning algorithms selection; (iii) data splitting into training and testing sets; (iv) data scaling; (v) hyper-parameters optimization by grid search with cross validation; and (vi) final model evaluation. These steps are detailed below. The ML models were compared with the hydrodynamic models of Wilke-Chang [5] and Tyn-Calus [7], the hybrid model of Zhu et al. [13] and one of the correlations proposed by Magalhães et al. [9].

### 2.1. Database Compilation

The database of binary diffusivities used in this work relied on the recent compilation published by Zêzere et al. [4], in the case of nonpolar solvent systems, and on an updated version of the database reported by Magalhães et al. for polar solvent systems [10]. Globally, database covers a wide range of temperatures (213.2–567.2 K) and densities (0.30–1.65 g cm^−3^) being composed by 244 binary systems and 2560 data points. This includes 90 polar systems (polar solvent/solute) totalizing 1431 points and 154 nonpolar systems (nonpolar solvent/solute) totalizing 1129 points. Data were collected for the 20 properties shown in Table 1. Whenever not reported by the authors, densities and viscosities were taken from the National Institute of Standards and Technology (NIST) database or calculated by the following set of equations: Yaws [26], Cibulka and Ziková [27], Cibulka et al. [28,29], Cibulka and Takagi [30], Przezdziecki and Sridhar [31], Viswanath et al. [32] the Lucas method [33], Assael et al. [34], Cano-Gómez et al. [35] and Pádua et al. [36]. Solute molar volumes at normal boiling point were estimated by Tyn–Calus equation [37]. The critical constants, whenever not provided with the diffusion data and not found in the other references [26,31,38,39,40,41,42,43,44], were estimated by Joback [31,45,46], Somayajulu [47], Klincewicz [31,48], Ambrose [31,49,50,51] and Wen–Qiang [52] methods. For ionic liquids the critical constants were retrieved from Valderrama and Rojas [53]. The acentric factors, when not provided, were estimated by the Lee-Kesler [54] and Pitzer [55] equations or retrieved from [26,31,38,39,40,41,42,43,44]. The Lennard-Jones diameter and energy were taken from Silva et al. [12] and, when not available, were estimated by equations 7 and 8 from Liu et al. [15] and equation 9 from Magalhães et al. [14]. Detailed information on the database used, including pure compound properties, is presented in Table 2.

Polar and nonpolar systems were separated into two databases based on the polarity of the solvent and, for each, data were split randomly 70/30% into training and testing sets. The training set was used for model learning and fitting, and the testing set was used to evaluate the performance of the fitted model after learning. Information from the testing set is never known during learning. In order to guarantee that all models are fed the same data, these data sets were also used for the evaluation of the classic models.

Most learning algorithms benefit from scaling input variables in order to improve model robustness and training speed [61]. The most common scaling strategies are normalization or standardization. Normalization consists in transforming the real range of values into a standard range (e.g., [0, 1] or [−1, 1]). Standardization consists in transforming variables so that they follow a standard normal distribution (mean of zero and standard deviation of one). In this work, variables were normalized to the [0, 1] range before passing them to training.

### 2.2. Variable Selection and Hyper-Parameter Optimization

Model variables were selected from the ones shown in Table 1 while removing collinear variables systematically. For each pair of variables with collinearity above a defined threshold of 0.50, the one with lower correlation with $D_{12}$ was removed from the model. The simplicity of obtaining a variable for a given system was also considered if both show similar correlation with $D_{12}$. This was done to improve the simplicity and ease of use of the final model.

Besides the model parameters discussed thus far, each learning algorithm possesses a set of parameters, which can be seen as configuration options, that specify how the algorithm behaves. These variables are often called hyper-parameters and are not fitted to data but rather must be set before training. Hyper-parameters were optimized for each learning algorithm using a grid search method with 4-fold cross validation implemented using *GridSearchCV* of *scikit-learn* (version 0.22.1). This method performs an exhaustive search for the best hyper-parameter values in a predefined grid by evaluating the model performance by 4-fold cross-validation. The *k*-fold cross-validation approach divides the training set into *k* subsets and trains the model with data from *k* − 1 of the folds while testing it on the fold. This process is repeated using every different *k* − 1 fold combination and the best model (best combination of hyper-parameters) emerges as that with the best average performance while avoiding both overfitting and underfitting of the models. The evaluated hyper-parameters for each learning algorithm are showed in Table S1 of the Supplementary Material.

### 2.3. Machine Learning Algorithms

Five ML algorithms were evaluated for the prediction of binary diffusivities: A multilinear regression, a *k*-nearest neighbors model, a decision tree algorithm, and two ensemble methods (random forest and gradient boosted). Models were implemented using the Python machine learning library *scikit-learn* version 0.22.1 [62].

A simple ordinary least squares multilinear regression was used as a baseline model for the prediction of binary diffusivities. In a multilinear regression [63], the target value, y, is a linear combination of explanatory variables, $x_{i}$, weighted by coefficients $b_{i}$. The coefficients are optimized to minimize the residual sum of squares between the observed and the calculated values. It was implemented using the *LinearRegression* class in *scikit-learn*.

The *k*-nearest neighbors (kNN) [64,65] is one of the simplest machine learning algorithms. Its prediction is the average of its *k* closest neighbors in the input space. Neighbors are selected from a set of examples for which the target property is known. This can be seen as the training set, although unlike other algorithms, kNN does not require an explicit training phase. The nearest neighbors are identified by position vectors in the multidimensional input space, usually in terms of Euclidean distance, nonetheless other distance measures could be applied. The kNN algorithm was implemented using the *KNeighborsRegressor* class in *scikit-learn*.

Decision tree [65,66] models take the training data and create a set of decision rules that are applied to the explanatory variables. Prediction is performed by following these tree-like rule graphs and selecting the paths that return the best metric, usually lowest entropy or largest information gain, until an output node is reached. The decision tree algorithm was implemented using the *DecisionTreeRegressor* class in *scikit-learn*.

Finally, ensemble methods are a combination of a large number of simple models, thus improving generalizability and robustness over a single model [63]. They can be divided into averaging ensemble methods, as the random forest algorithm, and boosting ensemble methods, such as the gradient boosted model, and have proven to be effective for regression learning [67].

Random forests [65,68] are comprised by several strong models, such as decision trees, trained independently. For the construction of each tree a random subset of training data is selected, while the remaining subset is used for testing. The final prediction is obtained as an average of the ensemble. Random forests are fast and simple to apply as they have simpler hyper-parameters settings than other methods, can be applied in cases with a large amount of noise and are less prone to overfitting [65]. The random forest model was implemented in *scikit-learn* using the *RandomForestRegressor* class.

Gradient boosted [69] models combine several learners, which are not independently trained but combined so that each new learner mitigates the bias of the previous one. The gradient boosted model also uses decision trees which are fitted to the gradient of a loss function, for instance, the squared error. The gradient is calculated for every sample of the training set but only a random subset of those gradients is used at by each learner. Gradient boosted has showed to provide very good predictions at least on par with random forests and usually superior to other methods [70]. The gradient boosted algorithm was implemented using the *GradientBoostingRegressor* class.

### 2.4. Classic Models

Several classic $D_{12}$ models were used as a benchmark for the proposed ML models, including the still extensively used Wilke-Chang equation [5], the Tyn-Calus equation [7], one of the Magalhães et al. correlations [9], and the Zhu et al. hybrid model [13]. Bellow, these models are briefly presented.

The Wilke-Chang equation [5] is an empirical modification of the Stokes-Einstein relation and is given by:

(1) $$D_{12}\left({\mathrm{cm}}^{2}s^{-1}\right)=\frac{7.4\times 10^{-8}{\left(\phi M_{1}\right)}^{0.5}T}{\mu_{1}{\left(V_{\mathrm{bp},2}\right)}^{0.6}}$$

where ϕ (dimensionless) is the association factor of the solvent (1.9 for the case of methanol, 1.5 for ethanol and 1.0 if it is unassociated [31]), and $V_{\mathrm{bp},2}$ (cm^3^ mol^−1^) is the solute molar volume at normal boiling temperature, which can be estimated using the critical volume ($V_{c,i}$) by the Tyn-Calus relation [31,37]:

(2) $$V_{\mathrm{bp},i}=0.285\times V_{c,i}^{1.048}$$

The Tyn-Calus equation [7] is another commonly used hydrodynamic equation, which is described by:

(3) $$D_{12}\left({\mathrm{cm}}^{2}s^{-1}\right)=8.93\times 10^{-8}\frac{V_{\mathrm{bp},1}^{0.267}}{V_{\mathrm{bp},2}^{0.433}}\frac{T}{\mu_{1}}$$

Magalhães et al. [9] proposed nine correlations for $D_{12}$, and four of them depend explicitly on solvent viscosity and temperature. Here we adopt the following:

(4) $$D_{12}=a\frac{T}{\mu_{1}}+b$$

where a and b are fitted parameters for each system. This equation consists of a modification of the Stokes–Einstein theory [31].

Zhu et al. [13] developed a hybrid model containing a component related with the free volume and another related with energy. It was devised for the estimation of $D_{12}$ of real nonpolar fluids. It is described by:

(5) $$\begin{array}{c} D_{12}=\frac{3}{8\sqrt{\pi }}\sqrt{\frac{\sigma_{\mathrm{LJ},12}^{2}\varepsilon_{\mathrm{LJ},12}}{m_{1}}}\frac{\sqrt{T_{12}^{*}}}{\rho_{12}^{*}}\left(1-\frac{\rho_{12}^{*}}{1.029079T_{12}^{*}{}^{0.165377}}\right)\\ \times \left[1+\rho_{12}^{*}{}^{0.126978}\left(\frac{0.596103\left(\rho_{12}^{*}-1\right)}{0.539292\left(\rho_{12}^{*}-1\right)+T_{12}^{*}{}^{\left(0.400152-0.41054\rho_{12}^{*}\right)}}+0.68856\right)\right]\\ \times exp\left(-\frac{\rho_{12}^{*}}{2T_{12}^{*}}\right) \end{array}$$

where the subscripts 1 and 2 denominate solvent and solute, respectively, $m_{1}$ is the mass of the solvent, and $\rho_{12}^{*}$ and $T_{12}^{*}$ are the density and temperature reduced using binary Lennard-Jones (LJ) parameters $\varepsilon_{\mathrm{LJ},12}$ and $\sigma_{\mathrm{LJ},12}$ as described by:

(6) $$T_{12}^{*}=T/\left(\varepsilon_{\mathrm{LJ},12}/k_{B}\right)$$

(7) $$\rho_{12}^{*}=\rho_{n,1}\sigma_{\mathrm{LJ},12}{}^{3}$$

The binary LJ parameters are calculated by the following combining rules:

(8) $$\sigma_{\mathrm{LJ},12}=\left(1-k_{12}^{d}\right)\frac{\left(\sigma_{\mathrm{LJ},2}+\sigma_{\mathrm{LJ},1}\right)}{2};\;\;\;\varepsilon_{\mathrm{LJ},12}/k_{B}=\sqrt{\left(\varepsilon_{\mathrm{LJ},1}/k_{B}\right)\left(\varepsilon_{\mathrm{LJ},2}/k_{B}\right)}$$

and the interaction parameter $k_{12}^{d}$ is estimated through:

(9) $$k_{12}^{d}=0.7926\frac{\left(\sigma_{\mathrm{LJ},2}-\sigma_{\mathrm{LJ},1}\right)}{\left(\sigma_{\mathrm{LJ},2}+\sigma_{\mathrm{LJ},1}\right)}$$

Finally, the LJ parameters $\varepsilon_{\mathrm{LJ}}/k_{B}$ and $\sigma_{\mathrm{LJ}}$ for the solute are calculated by:

(10) $$\varepsilon_{\mathrm{LJ},2}/k_{B}=T_{c,2}/1.313;\;\;\;\;\;\;\;\;\;\;\sigma_{\mathrm{LJ},2}=\left(0.13\varepsilon_{\mathrm{LJ},2}/P_{c,2}\right)$$

and for the solvent:

(11) $$\varepsilon_{\mathrm{LJ},1}/k_{B}=T_{c,1}/1.313\left(1+0.47527332\rho_{r,1}+\left(0.06300484+0.12374707\rho_{r,1}\right)T_{r,1}\right)$$

(12) $$\sigma_{\mathrm{LJ},1}={\left(0.31/\rho_{n,c,1}\right)}^{1/3}\left(1-0.0368868\rho_{r,1}+\left(0.00006945+0.01089228\rho_{r,1}\right)T_{r,1}\right)$$

where $\rho_{n,c,1}$ is the number critical density (cm^−3^) and $\rho_{r,1}$ and $T_{r,1}$ are the reduced density and reduced temperature of the solvent, calculated with the corresponding critical constants: $P_{r,1}=P_{1}/P_{c,1}$ and $T_{r,1}=T_{1}/T_{c,1}$.

## 3. Results and Discussion

### 3.1. Machine Learning Models

**Figure 1:**
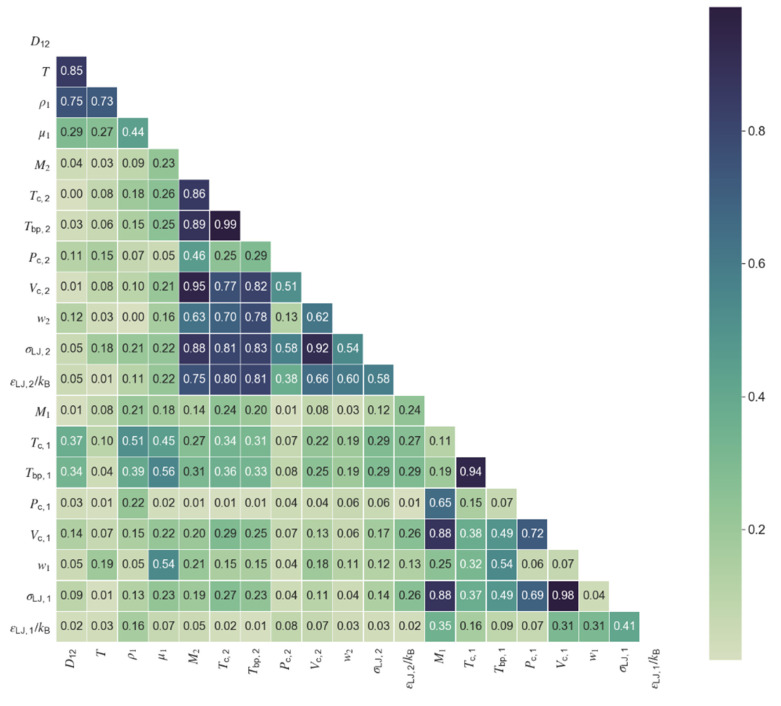
Correlation heat map for all properties and variables in the database of polar compounds. Colormap shows the absolute value of the Pearson correlation from zero (light green) to one (dark blue).

**Figure 2:**
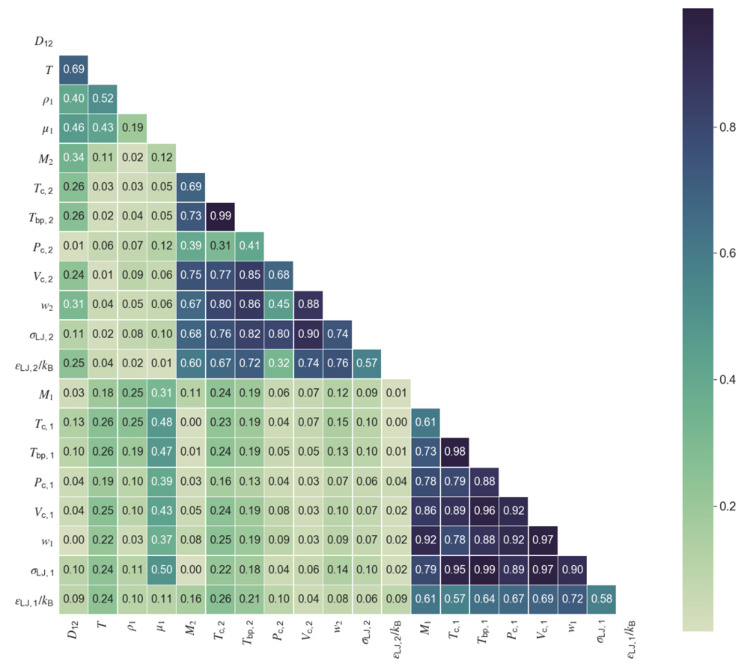
Correlation heat map for all properties and variables in the database of nonpolar compounds. Colormap shows the absolute value of the Pearson correlation from zero (light green) to one (dark blue).

The first step towards model development was the choice of relevant variables for the model. Selection was conducted on the basis of the collinearities between the available variables/properties and their level of correlation with the diffusivity. Figure 1 and Figure 2 show the correlation matrix (in the form of a heat map) for the polar and nonpolar data sets, where the values represent the absolute Pearson correlation. When two variables presented collinearities above a defined threshold of 0.50, only one was kept in the model, namely the one providing of the best correlation with diffusivity. Following this procedure, six variables were selected for the polar diffusivity model: temperature, solvent viscosity, solute molar mass, solute critical pressure, solvent molar mass, and the Lennard-Jones energy constant of solvent. For the nonpolar diffusivity model, temperature, solvent viscosity, solute molar mass, solute critical pressure, and solvent molar mass were chosen, totaling five variables. A summary of the variables required for the machine learning models for polar (ML Polar) and nonpolar (ML Nonpolar) systems is presented in Table 3, together with the required inputs for the classic models of Wilke-Chang, Tyn-Calus, Magalhães et al., and Zhu et al. The two hydrodynamic equations require four input variables, the same number as the Magalhães et al. correlation although, in this later case, two of the four parameters must be fitted to experimental data, thus reducing the model applicability. The Zhu et al. hybrid model requires the larger number of parameters (seven) and is only applicable to nonpolar systems.

The performance of all models was evaluated by calculating the average absolute relative deviation (AARD) of each system:

(13) $$\mathrm{AARD}\left(\%\right)=\frac{100}{\mathrm{NDP}}\overset{\mathrm{NDP}}{\underset{i=1}{\sum }}{\left|\frac{D_{12,i}^{\mathrm{calc}}-D_{12,i}^{\exp}}{D_{12,i}^{\exp}}\right|}_{\;}$$

where superscripts calc and exp denote calculated and experimental values, and NDP is the number of data points of a system. For the whole database, the global deviation (i.e., weighted AARD) and the arithmetic systems average (AARD_arith_) were calculated. The minimum and maximum system AARD are reported as an indication of the performance of the best and worst systems. The root mean square error (RMSE) was also calculated and is defined as:

(14) $$\mathrm{RMSE}=\sqrt{\frac{1}{\mathrm{NDP}}\overset{\mathrm{NDP}}{\underset{i=1}{\sum }}{\left(D_{12,i}^{\mathrm{calc}}-D_{12,i}^{\exp}\right)}_{\;}{}^{2}}$$

The coefficient of determination, *R*^2^, which is calculated for the training set, and the *Q*^2^ value, which corresponds to *R*^2^ value obtained when applying the model to the test set, are also reported for all models.

**Figure 3:**
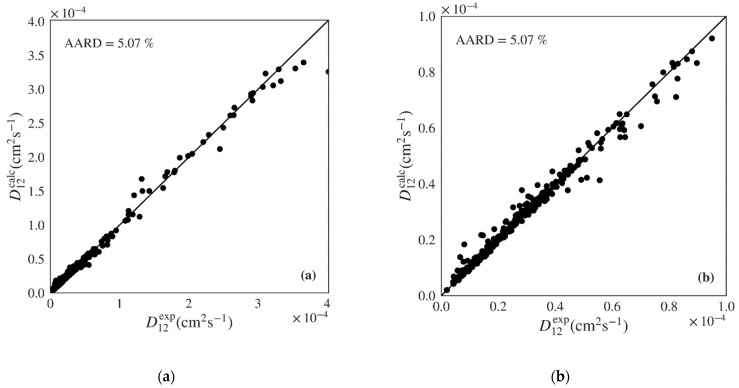
Predicted *versus* experimental diffusivities for the test set of polar systems for the best machine learning model (Gradient Boosted): (**a**) plot including all calculated results; (**b**) plot zooming on lower $D_{12}$ range.

A final validation of the best machine learning models was conducted by performing a y-randomization test (also called y-scrambling). This test compares the performance of the original model with that of models built for a scrambled (randomly shuffled) response while still following the original model building procedure. The randomization process eliminates the relation between the independent variables and target response. If the performance of the models when using scrambled data is much lower than when using original data, one can be confident of the relevance of the original model. Five algorithms were tested to develop the supervised learning models including a multilinear regression, *k*-nearest neighbors, decision tree, random forest (an averaging ensemble method), and gradient boosted (a boosting ensemble method). The performance of the several machine learning algorithms when applied to the test set of polar data, covering 79 systems and 430 points, is shown in Table 4. The gradient boosted algorithm presents the best performance for the test set (pure prediction) with an AARD of 5.07% followed by the random forest, decision tree, *k*-nearest neighbors, and multilinear regression (from lower to higher AARD). Similar trends are present when analyzing the arithmetic average of 79 systems AARD, as well as the minimum and the maximum AARD. As expected, the multilinear regression exhibits much worse results than the other four algorithms for all the AARD metrics. The gradient boosted algorithm also presents the lowest RMSE and highest *Q*^2^. The *Q*^2^ value is also close to *R*^2^ indicating that the model works well independently of its training data. Figure 3 plots the diffusivities predicted by the gradient boosted ML model against the respective experimental values for the test set of polar systems, showing a very good distribution along the diagonal. Similar representations are provided for the remaining four algorithms in Figures S1–S4 of the Supplementary Material. The multilinear regression model presents significant underestimation at higher values of $D_{12}$ and overestimation in the intermediate region. On the other hand, the remaining three algorithms show good dispersion around the diagonal, however with larger deviations than the gradient boosted model.

**Figure 4:**
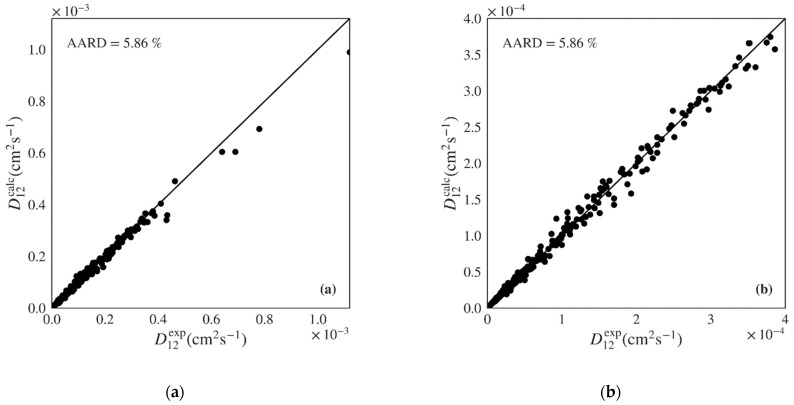
Predicted *versus* experimental diffusivities for the test set of nonpolar systems for the best machine learning model (Gradient Boosted) showing (**a**) plot including all calculated results; (**b**) plot zooming on lower $D_{12}$ range.

Table 5 presents the results obtained using each ML algorithm for the test set of nonpolar compounds (130 systems and 342 points). Once again, the gradient boosted algorithm presents the best global AARD for the 130 systems of the test set (5.86%), followed by the random forest, then by the decision tree and *k*-nearest neighbors with similar results, and lastly by the multilinear regression with significantly worst results. A similar trend is visible when calculating a simple arithmetic average of systems AARD. The gradient boosted algorithm shows the lowest RMSE and highest *Q*^2^. The calculated *versus* experimental diffusivities for the test set of nonpolar compounds using the Gradient Boosted model are plotted in Figure 4, showing unbiased distribution along the diagonal over all range of experimental points. Figures S5–S8 of the Supplementary Material provide the calculated against experimental plots for the remaining four algorithms. As in the case of the polar data, the multilinear regression model once again presents significant deviations. The *k*-nearest neighbors, decision tree, and random forest algorithms provide better scattering around the diagonal. Few outliers may be observed, particularly in the case of the decision tree model.

As a final validation of the gradient boosted models selected for polar and nonpolar systems, a y-randomization test was performed by scrambling the diffusivity vector. This process was repeated 200 times and always returned random models with performances much lower than the original ones, thus confirming the significance of the proposed models. Figure S9 of the Supplementary Material shows the contrast between the *Q*^2^ values of our models (0.9919 for polar and 0.9879 for nonpolar) and the lower ones obtained for the permutations. It is worth noting that: (i) the best possible score of *Q*^2^ (and *R*^2^) is 1.0; (ii) for a constant model that always predicts the expected value of the response, both indicators are zero; (iii) *Q*^2^ (and *R*^2^) can be negative for arbitrarily worse model.

Summarily, the ML Polar Gradient Boosted model showed good performance for the prediction of diffusivities of multiple solutes in polar solvents in the following train and test domain: T = 268–554 K; $\mu_{1}$ = 0.0241–17.6 cP; $M_{2}$ = 17–674 g mol^−1^; $P_{c,2}$ = 4.1–221.2 bar; $M_{1}$ = 20–113 g mol^−1^; and $\varepsilon_{\mathrm{LJ},1}/k_{B}$ = 208–2121 K. Likewise the ML Nonpolar Gradient Boosted can be applied over: T = 213–567 K; $\mu_{1}$ = 0.0229–2.92 cP; $M_{2}$ = 2–461 g mol^−1^; $P_{c,2}$ = 12.5–96.3 bar; and $M_{1}$ = 30–395 g mol^−1^. Both models showed good interpolation capability, however it is expected that they can also provide reasonable extrapolations.

The ML Polar Gradient Boosted and ML Nonpolar Gradient Boosted models are provided as a command line program in the Supplementary Material.

### 3.2. Detailed Comparison of ML Gradient Boosted and Classic Models

Four classic models for the calculation of diffusivities were adopted for comparison: two hydrodynamic equations (Wilke-Chang [5] and Tyn-Calus [7]), a correlation by Magalhães et al. [9], and the hybrid model of Zhu et al. [13]. The performance metrics of the classic models are shown in Table 4, for the polar systems, and Table 5, for the nonpolar systems. Overall, the proposed ML models outperform the classic models.

**Figure 5:**
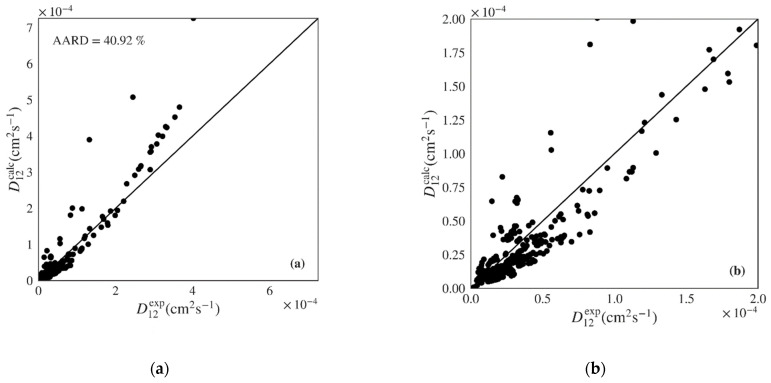

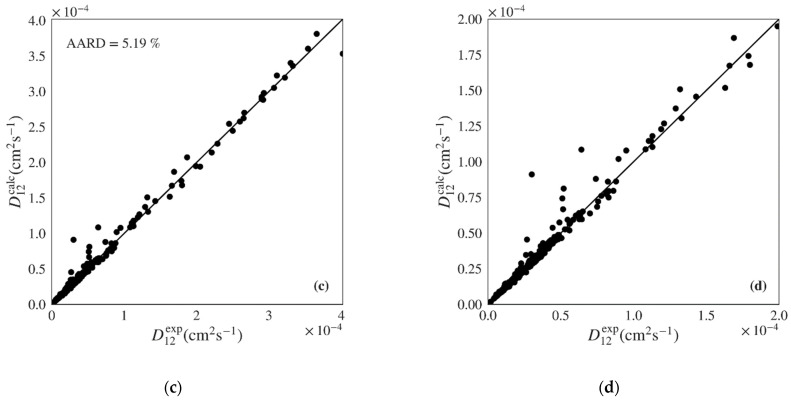
Calculated *versus* experimental diffusivities for the test set of polar systems for: (**a**) and (**b**) Wilke-Chang (Equation (1)) [5] and (**c**) and (**d**) Magalhães et al. (Equation (4)) [9] models. Note the distinct scale between plots.

**Figure 6:**
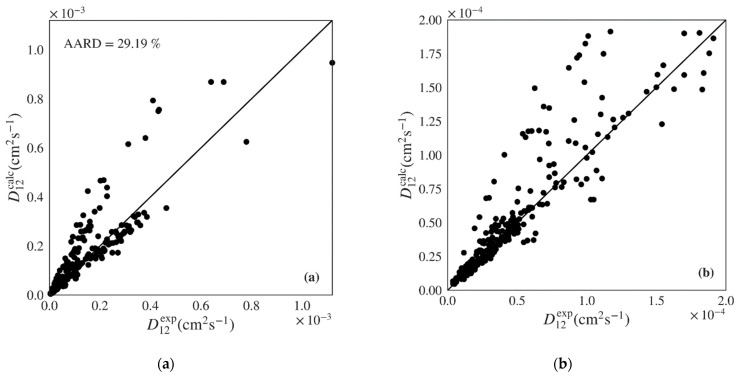

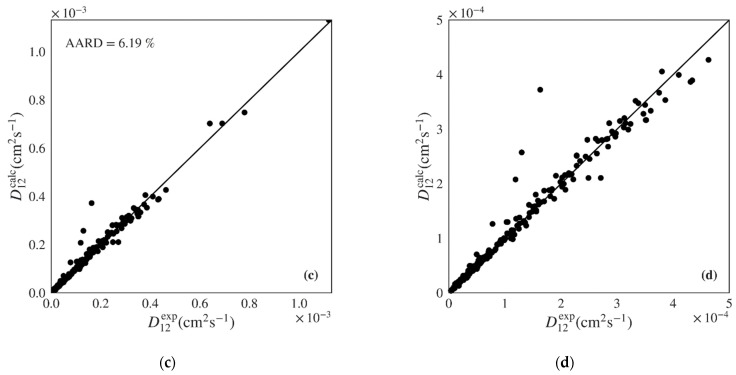
Calculated *versus* experimental diffusivities for the test set of nonpolar systems for: (**a**) and (**b**) Wilke-Chang (Equation (1)) [5] and (**c**) and (**d**) Magalhães et al. (Equation (4)) [9] models. Note the distinct scale between plots.

The Wilke-Chang and Tyn-Calus hydrodynamic equations provide similar performance indicators in both data sets, though the former shows much higher maximum AARDs (Table 4: 197.71% vs. 97.11%; Table 5: 172.30% vs. 64.97%). Analyzing Figure 5a,b, where the calculated *versus* experimental diffusivities are plotted for the polar data set over the entire range and over a low range of values, we see that the Wilke-Chang equation overestimates higher diffusivities and tends to underestimate lower ones. The Tyn-Calus equation for polar solvents provides systematic underestimation as shown in Figure S10 of the Supplementary Material. In the case of nonpolar systems, both Wilke-Chang (Figure 6a,b) and Tyn-Calus (Figure S11) models exhibit a dual biased distribution of the calculated $D_{12}$ values.

The correlation of Magalhães et al. is able to deliver the best performance among the classic models, with a unbiased distribution along the diagonal in Figure 5c,d and Figure 6c,d and an AARD only slightly above that provided by the machine learning gradient boosted models proposed in this work (5.19% and 6.19% for the polar and nonpolar sets, respectively). However, the Magalhães et al. can often be difficult to apply since it requires that data on the system of interest is available in order to fit its two parameters. In this work, data in the train sets was used to fit the a and b parameters for each system, which were then applied to the calculation of diffusivities for the test sets. For this reason, fewer points were calculated for the Magalhães et al. model, corresponding to the systems where not enough data were available in the train sets to optimize parameters a and b.

Finally, the Zhu et al. model, which was developed for nonpolar and weakly polar fluids, does not appear to provide any benefit over the much simpler Wilke-Chang and Tyn-Calus equations when applied to the nonpolar data set of this work. It provides higher AARD (Table 5: 37.93%) than both hydrodynamic equations (Table 5: 29.19% and 28.84%, respectively), although it shows lower biased dispersion along diagonal (Figure S12).

Table 6 details the results of the best machine learning (gradient boosted) and classic diffusivity models for each system of the polar database, as well as the distribution of points among train and test sets. The best results are found for the ethylbenzene/acetone system (AARD of 0.08%) and the worst for the ethylene glycol/ethanol system (76.23%). However, these two systems have only one and two points in the test set, respectively. Considering only cases where at least 10 points are available for train and test sets, the carbon dioxide/n-butanol shows the best result (1.19%) while ammonia/1-propanol has the worst (5.65%).

Table 7 presents equivalent information for the nonpolar systems. In this case, the *n*-decane/*n*-dodecane and tetraethyltin/*n*-decane systems show the best (0.03%) and worst (25.87%) results, respectively, but, once again, with only one point in the test set. If only systems with at least five points in the train and test sets are considered, the best result appears for 1,3,5-trimethylbenzene/*n*-hexane (2.98%) and the worst for toluene/n-hexane (4.58%).

The models proposed in this work can be easily retrained as new experimental data is made available, thus increasing its robustness and scope. A program that allows the estimation of diffusivities in polar and nonpolar systems is provided in the Supplementary Material, along with instructions on its use.

## 4. Conclusions

Two machine learning (ML) models were developed for the estimation of binary diffusivities in polar and nonpolar systems. These models were trained and tested on a database containing 20 properties for polar (90 systems and 1431 points) and nonpolar (154 systems and 1129 points) systems. Several learning algorithms were tested, including multilinear regression, *k*-nearest neighbors, decision tree, random forest and gradient boosted. The best ML results were obtained for the gradient boosted model, which provided global AARDs of 5.07% and 5.86% for the test set of polar and nonpolar systems, respectively. The nonpolar model relies on five input variables/properties: temperature, solvent viscosity, solute molar mass, solute critical pressure and solvent molar mass. The polar model takes the Lennard-Jones energy of solvent as an additional parameter, thus requiring six inputs totally. The classic models of Wilke-Chang, Tyn-Calus, Magalhães et al. and Zhu et al. were adopted for comparison and demonstrated worse performance for the same test sets. The 2-parameter correlation of Magalhães et al. showed results closer to the new gradient boosted models with AARD of 5.19% (polar) and 6.19% (nonpolar), however, that equation requires previous data to fit its two parameters, and thus it is impractical to apply to unknown systems. Among the remaining classic models, Wilke-Chang provided the best result for polar systems (40.92%) while Tyn-Calus performed best for nonpolar systems (28.84%). The developed models are provided as application in the Supplementary Material.

## Figures and Tables

**Table 1:** Properties and variables available for each system in the database.

Property	Units	Description
$D_{12}$	cm^2^ s^−1^	Diffusion coefficient
T	K	Temperature
$\rho_{1}$	g cm^−3^	Solvent density
$\mu_{1}$	cP	Solvent viscosity
$M_{1}$	g mol^−1^	Molar mass of solvent
$M_{2}$	g mol^−1^	Molar mass of solute
$T_{c,1}$	K	Critical temperature of solvent
$T_{c,2}$	K	Critical temperature of solute
$T_{\mathrm{bp},1}$	K	Normal boiling point temperature of solvent
$T_{\mathrm{bp},2}$	K	Normal boiling point temperature of solute
$P_{c,1}$	bar	Critical pressure of solvent
$P_{c,2}$	bar	Critical pressure of solute
$V_{c,1}$	cm^3^ mol^−1^	Critical volume of solvent
$V_{c,2}$	cm^3^ mol^−1^	Critical volume of solute
$w_{1}$	-	Acentric factor of solvent
$w_{2}$	-	Acentric factor of solute
$\sigma_{\mathrm{LJ},1}$	Å	Lennard-Jones diameter of solvent
$\sigma_{\mathrm{LJ},2}$	Å	Lennard-Jones diameter of solute
$\varepsilon_{\mathrm{LJ},1}/k_{B}$	K	Lennard-Jones energy constant of solvent
$\varepsilon_{\mathrm{LJ},2}/k_{B}$	K	Lennard-Jones energy constant of solute

**Table 2:** Pure compounds properties and respective sources.

Compound	Formula	CAS	M (g mol^−1^)	$T_{c}$ (K)	$T_{b}$ (K)	$P_{c}$ (bar)	$V_{c}$ (cm^3^ mol^−1^)	w	$\sigma_{LJ}$ (Å)	$\varepsilon_{LJ}/k_{B}$ (K)
[Bmim][bti]	C_10_H_15_N_3_F_6_S_2_O_4_	174899-83-3	419.40	1269.90 ^a^	862.40 ^a^	27.60 ^a^	990.10 ^a^	0.3004 ^a^	7.59636 ^t^	982.90 ^t^
[Emim][bti]	C_8_H_11_N_3_F_6_S_2_O_4_	174899-82-2	391.31	1249.30 ^a^	816.70 ^a^	32.70 ^a^	875.90 ^a^	0.2157 ^a^	7.23444 ^t^	966.96 ^t^
[Hmim][bti]	C_12_H_19_N_3_F_6_S_2_O_4_	382150-50-7	447.42	1292.80 ^a^	908.20 ^a^	23.90 ^a^	1104.40 ^a^	0.3893 ^a^	7.90445 ^t^	1000.63 ^t^
[Omim][bti]	C_14_H_23_N_3_F_6_S_2_O_4_	862731-66-6	475.50	1317.80 ^a^	954.00 ^a^	21.00 ^a^	1218.60 ^a^	0.4811 ^a^	8.17464 ^t^	1019.98 ^t^
1,1-dimethylferrocene	C_12_H_14_Fe	1291-47-0	214.09	514.45 ^b^	353.55 ^c^	27.41 ^b^	400.64 ^b^	0.3453 ^d^	5.88660 ^t^	398.18 ^t^
1,2,3,5-tetrafluorobenzene	C_6_H_2_F_4_	2367-82-0	150.08	555.49 ^e^	375.38 ^f^	36.40 ^e^	351.05 ^e^	0.3817 ^d^	5.52349 ^t^	429.95 ^t^
1,2,4,5-tetrafluorobenzene	C_6_H_2_F_4_	327-54-8	150.08	535.25 ^g^	357.61 ^g^	37.47 ^g^	351.05 ^e^	0.3437 ^d^	5.41106 ^t^	414.28 ^t^
1,2,4-trichlorobenzene	C_6_H_3_Cl_3_	120-82-1	181.45	725.00 ^h^	486.15 ^h^	37.20 ^h^	395.00 ^h^	0.3580 ^h^	5.95446 ^t^	561.15 ^t^
1,2,4-trifluorobenzene	C_6_H_3_F_3_	367-23-7	132.09	558.22 ^e^	371.13 ^f^	38.98 ^e^	335.05 ^e^	0.3377 ^d^	5.41530 ^t^	432.06 ^t^
1,2-butanediol	C_4_H_10_O_2_	584-03-2	90.12	622.14 ^h^	463.46 ^h^	50.30 ^h^	291.50 ^h^	1.1410 ^h^	5.17223 ^t^	481.54 ^t^
1,3,5-trimethylbenzene	C_9_H_12_	108-67-8	120.20	637.30 ^i^	437.90 ^i^	31.30 ^i^	433.00 ^h^	0.3990 ^i^	6.03392 ^t^	493.27 ^t^
1,3-dibromobenzene	C_6_H_4_Br_2_	108-36-1	235.91	761.00 ^h^	491.15 ^h^	46.60 ^h^	372.00 ^h^	0.2930 ^h^	5.64056 ^t^	589.01 ^t^
1,4-butanediol	C_4_H_10_O_2_	111-63-4	90.12	667.00 ^h^	501.15 ^h^	48.80 ^h^	297.00 ^h^	1.1890 ^h^	5.33697 ^t^	516.26 ^t^
12-crown-4	C_8_H_16_O_4_	294-93-9	176.21	780.66 ^e^	540.08 ^f^	33.59 ^e^	444.75 ^e^	0.4598 ^d^	6.27811 ^t^	604.23 ^t^
15-crown-5	C_10_H_20_O_5_	33100-27-5	220.27	876.80 ^e^	625.60 ^f^	28.72 ^e^	548.75 ^e^	0.5562 ^d^	6.79750 ^t^	678.64 ^t^
18-crown-6	C_12_H_24_O_6_	17455-13-9	264.32	970.51 ^e^	711.12 ^f^	24.95 ^e^	652.75 ^e^	0.6510 ^d^	7.26959 ^t^	751.17 ^t^
1-butanol	C_4_H_10_O	71-36-3	74.12	563.10 ^i^	390.90 ^i^	44.20 ^i^	275.00 ^i^	0.5930 ^i^	5.22056 ^t^	435.84 ^t^
1-octene	C_8_H_16_	111-66-0	112.22	566.70 ^i^	394.40 ^i^	26.20 ^i^	464.00 ^i^	0.3860 ^i^	6.14478 ^t^	438.63 ^t^
1-propanol	C_3_H_8_O	71-23-8	60.10	536.80 ^i^	370.30 ^i^	51.70 ^i^	219.00 ^i^	0.6230 ^i^	4.49190 ^u^	2120.83 ^u^
1-tetradecene	C_14_H_28_	1120-36-1	196.37	691.00 ^j^	524.25 ^j^	16.27 ^j^	865.00 ^j^	0.6503 ^j^	7.44105 ^t^	534.83 ^t^
2,2,4-trimethylpentane	C_8_H_18_	540-84-1	144.23	543.80 ^h^	372.39 ^h^	25.70 ^h^	468.00 ^h^	0.3030 ^h^	6.10433 ^t^	420.90 ^t^
2,3-dimethylbutane	C_6_H_14_	79-29-8	86.18	500.00 ^i^	331.10 ^i^	31.30 ^i^	358.00 ^i^	0.2470 ^i^	5.60227 ^t^	387.00 ^t^
2-phenylethyl acetate	C_10_H_12_O_2_	103-45-7	164.10	712.23 ^k^	505.16 ^f^	30.12 ^k^	524.15 ^k^	0.5442 ^d^	6.31046 ^t^	551.27 ^t^
2-propanol	C_3_H_8_O	67-63-0	60.10	508.30 ^i^	355.40 ^i^	47.60 ^i^	220.00 ^i^	0.6650 ^i^	4.93749 ^t^	393.42 ^t^
3-phenylpropyl acetate	C_11_H_14_O_2_	122-72-5	178.30	718.70 ^k^	518.16 ^f^	27.23 ^k^	580.37 ^k^	0.5924 ^d^	6.51801 ^t^	556.27 ^t^
9,10-dimethylanthracene	C_16_H_14_	781-43-1	206.29	899.22 ^e^	645.06 ^f^	26.27 ^e^	724.55 ^e^	0.5451 ^d^	7.01984 ^t^	696.00 ^t^
acetone	C_3_H_6_O	67-64-1	58.08	508.10 ^i^	329.20 ^i^	47.00 ^i^	209.00 ^i^	0.3040 ^i^	4.67012 ^u^	332.97 ^u^
acetonitrile	C_2_H_3_N	75-05-8	41.05	545.50 ^i^	354.80 ^i^	48.30 ^i^	173.00 ^i^	0.3270 ^i^	4.02424 ^u^	652.53 ^u^
acridine	C_13_H_9_N	260-94-6	179.22	905.00 ^l^	619.15 ^l^	36.40 ^l^	543.00 ^l^	0.4381 ^d^	6.40475 ^t^	700.47 ^t^
ammonia	NH_3_	7664-41-7	17.03	405.50 ^i^	239.80 ^i^	113.30 ^i^	72.50 ^i^	0.2500 ^i^	4.24397 ^u^	4.46 ^u^
argon	Ar	7440-37-1	39.95	150.80 ^i^	87.30 ^i^	48.70 ^i^	74.90 ^i^	0.0010 ^i^	3.40744 ^u^	123.55 ^u^
astaxanthin	C_40_H_52_O_4_	472-61-7	596.84	1148.51 ^f^	1047.00 ^f^	5.30 ^f^	1877.50 ^f^	2.8439 ^d^	9.98026 ^t^	888.95 ^t^
benzene	C_6_H_6_	71-43-2	78.11	562.20 ^i^	353.20 ^i^	48.90 ^i^	259.00 ^i^	0.2120 ^i^	5.19165 ^u^	308.43 ^u^
benzoic acid	C_7_H_6_O_2_	65-85-0	122.12	752.00 ^i^	523.00 ^i^	45.60 ^i^	341.00 ^i^	0.6200 ^i^	5.65763 ^t^	582.05 ^t^
benzonitrile	C_7_H_5_N	100-47-0	103.12	699.35 ^h^	464.15 ^h^	42.15 ^h^	339.00 ^h^	0.3520 ^h^	5.66827 ^t^	541.30 ^t^
benzothiophene	C_8_H_6_S	95-15-8	134.20	764.00 ^j^	494.05 ^j^	47.60 ^j^	379.00 ^j^	0.3071 ^j^	5.61049 ^t^	591.34 ^t^
benzyl acetate	C_9_H_10_O_2_	140-11-4	150.18	699.00 ^h^	486.65 ^h^	31.80 ^h^	449.00 ^h^	0.4700 ^h^	6.17454 ^t^	541.03 ^t^
biphenyl	C_12_H_10_	92-52-4	154.21	789.00 ^i^	529.30 ^i^	38.50 ^i^	502.00 ^i^	0.3720 ^i^	6.04576 ^t^	610.69 ^t^
carbon dioxide	CO_2_	124-38-9	44.01	304.10 ^i^	194.70 ^h^	73.80 ^i^	93.90 ^i^	0.2390 ^i^	3.26192 ^u^	500.71 ^u^
carbon disulfide	CS_2_	75-15-0	76.13	552.00 ^i^	319.00 ^i^	79.00 ^i^	160.00 ^i^	0.1090 ^i^	4.29901 ^u^	376.51 ^u^
carbon monoxide	CO	630-08-0	28.01	132.90 ^i^	81.70 ^i^	35.00 ^i^	93.20 ^i^	0.0660 ^i^	3.53562 ^t^	102.86 ^t^
carbon tetrabromide	CBr_4_	558-13-4	331.63	724.91 ^h^	462.65 ^h^	96.31 ^h^	328.50 ^h^	0.5010 ^h^	4.41501 ^t^	561.08 ^t^
carbon tetrachloride	CCl_4_	56-23-5	153.82	556.40 ^i^	349.90 ^i^	45.60 ^i^	275.90 ^i^	0.1930 ^i^	5.29240 ^u^	418.84 ^u^
chlorobenzene	C_6_H_5_Cl	108-90-7	112.56	632.40 ^i^	404.90 ^i^	45.20 ^i^	308.00 ^i^	0.2490 ^i^	5.56838 ^u^	207.50 ^u^
chlorotrifluoromethane	CClF_3_	75-72-9	104.46	302.00 ^i^	193.20 ^i^	38.70 ^i^	180.40 ^i^	0.1980 ^i^	4.37636 ^u^	410.79 ^u^
chromium(III) acetylacetonate	Cr(acac)_3_	21679-31-2	349.32	858.85 ^b^	613.15 ^m^	18.92 ^b^	627.04 ^b^	0.3631 ^d^	5.71650 ^v^	845.60 ^v^
cyclohexane	C_6_H_12_	110-82-7	84.16	553.50 ^i^	353.80 ^i^	40.70 ^i^	308.00 ^i^	0.2120 ^i^	5.73075 ^u^	224.87 ^u^
deuterium oxide	D_2_O	7789-20-0	20.03	643.89 ^i^	374.55 ^i^	216.71 ^i^	56.26 ^i^	0.3447 ^d^	3.26304 ^t^	498.37 ^t^
dibenzothiophene	C_12_H_8_S	132-65-0	184.26	897.00 ^j^	604.61 ^j^	38.60 ^j^	512.00 ^j^	0.3983 ^j^	6.27791 ^t^	694.28 ^t^
dibenzyl ether	C_14_H_14_O	103-50-4	198.27	777.00 ^h^	561.45 ^h^	25.60 ^h^	608.00 ^h^	0.5910 ^h^	6.78621 ^t^	601.40 ^t^
dicyclohexano-18-crown-6	C_20_H_36_O_6_	16069-36-6	372.50	1177.47 ^e^	906.84 ^f^	16.24 ^e^	1002.75 ^e^	0.7675 ^d^	8.41774 ^t^	911.36 ^t^
dicyclohexano-24-crown-8	C_24_H_44_O_8_	17455-23-1	460.61	1357.66 ^e^	1077.88 ^f^	13.48 ^e^	1210.75 ^e^	0.9120 ^d^	8.62250 ^t^	1050.83 ^t^
disperse blue 14	C_16_H_14_N_2_O_2_	2475-44-7	266.00	1137.33 ^f^	881.88 ^f^	27.18 ^f^	765.50 ^f^	1.1790 ^d^	7.41187 ^t^	880.29 ^t^
disperse orange 11	C_15_H_11_NO_2_	82-28-0	237.25	1103.62 ^f^	831.19 ^f^	31.17 ^f^	670.00 ^f^	0.9859 ^d^	7.08580 ^t^	854.20 ^t^
ethane	C_2_H_6_	74-84-0	30.07	305.40 ^i^	184.60 ^i^	48.80 ^i^	148.30 ^i^	0.0990 ^i^	4.17587 ^u^	213.99 ^u^
ethanol	C_2_H_6_O	64-17-5	46.07	513.90 ^i^	351.40 ^i^	61.40 ^i^	167.10 ^i^	0.6440 ^i^	4.23738 ^u^	1291.41 ^u^
ethyl acetate	C_4_H_8_O_2_	141-78-6	88.11	523.20 ^i^	350.30 ^i^	38.30 ^i^	286.00 ^i^	0.3620 ^i^	5.33606 ^t^	404.96 ^t^
ethylbenzene	C_8_H_10_	100-41-4	106.17	617.20 ^i^	409.30 ^i^	36.00 ^i^	374.00 ^i^	0.3020 ^i^	5.72572 ^t^	477.71 ^t^
ethylene	C_2_H_4_	74-85-1	28.05	282.40 ^i^	169.30 ^i^	50.40 ^i^	130.40 ^i^	0.0890 ^i^	4.04838 ^u^	169.08 ^u^
ethylene glycol	C_2_H_6_O_2_	107-21-1	62.07	645.00 ^h^	470.45 ^h^	75.30 ^h^	191.00 ^h^	1.1907 ^d^	4.60221 ^t^	499.23 ^t^
ethylferrocene	C_12_H_14_Fe	1273-89-8	214.08	554.21 ^b^	381.75 ^n^	27.41 ^b^	400.64 ^b^	0.3556 ^d^	6.02127 ^t^	428.96 ^t^
eucalyptol	C_10_H_18_O	470-82-6	154.25	695.50 ^o^	449.50 ^f^	31.40 ^o^	509.50 ^o^	0.6490 ^b^	6.18868 ^t^	538.32 ^t^
ferrocene	C_10_H_10_Fe	102-54-5	186.04	786.27 ^b^	522.15 ^n^	32.07 ^b^	317.77 ^b^	0.2638 ^d^	6.37838 ^t^	608.57 ^t^
gallic acid	C_7_H_6_O_5_	149-91-7	170.12	1136.70 ^p^	789.90 ^p^	34.90 ^p^	276.20 ^p^	0.4984 ^d^	6.92304 ^t^	879.81 ^t^
glycerol	C_3_H_8_O_3_	56-81-5	92.10	723.00 ^h^	563.15 ^h^	40.00 ^h^	264.00 ^h^	1.4986 ^d^	5.81929 ^t^	559.60 ^t^
hexafluorobenzene	C_6_F_6_	392-56-3	186.06	516.70 ^i^	353.40 ^i^	33.00 ^i^	335.00 ^i^	0.3960 ^i^	5.56763 ^t^	399.93 ^t^
hydrogen	H_2_	1333-74-0	2.02	33.00 ^i^	20.30 ^i^	12.90 ^i^	64.30 ^i^	−0.2160 ^i^	5.94111 ^u^	0.00 ^u^
Ibuprofen	C_13_H_18_O_2_	15687-27-1	206.29	769.63 ^e^	580.45 ^q^	22.85 ^e^	686.35 ^e^	0.8512 ^d^	6.98841 ^t^	595.69 ^t^
indole	C_8_H_7_N	204-420-7	117.15	790.00 ^h^	526.15 ^h^	43.40 ^h^	431.00 ^h^	0.4293 ^y^	5.83184 ^t^	611.46 ^t^
krypton	Kr	7439-90-9	83.80	209.40 ^i^	119.90 ^i^	55.00 ^i^	91.20 ^i^	0.0050 ^i^	2.89870 ^u^	511.92 ^u^
linoleic acid methyl ester	C_19_H_34_O_2_	112-63-0	294.48	870.78 ^r^	700.66 ^f^	12.54 ^r^	1070.95 ^r^	0.9952 ^d^	8.34769 ^t^	673.98 ^t^
methane	CH_4_	74-82-8	16.04	190.40 ^i^	111.60 ^i^	46.00 ^i^	99.20 ^i^	0.0110 ^i^	3.58484 ^u^	167.15 ^u^
methanol	CH_4_O	67-56-1	32.04	512.60 ^i^	337.70 ^i^	80.90 ^i^	118.00 ^i^	0.5560 ^i^	3.79957 ^u^	685.96 ^u^
*m*-xylene	C_8_H_10_	108-38-3	106.17	617.10 ^i^	412.30 ^i^	35.40 ^i^	376.00 ^i^	0.3250 ^i^	5.75507 ^t^	477.64 ^t^
naphthalene	C_10_H_8_	91-20-3	128.17	748.40 ^i^	491.10 ^i^	40.50 ^i^	413.00 ^i^	0.3020 ^i^	5.85874 ^t^	579.26 ^t^
*n*-butanol	C_410_O	71-36-3	74.12	563.10 ^i^	390.90 ^i^	44.20 ^i^	275.00 ^i^	0.5930 ^i^	5.22056 ^t^	435.84 ^t^
*n*-decane	C_10_H_22_	124-18-5	142.29	617.70 ^i^	447.30 ^i^	21.20 ^i^	603.00 ^i^	0.4890 ^i^	6.71395 ^u^	434.86 ^u^
*n*-dodecane	C_12_H_26_	112-40-3	170.34	658.20 ^i^	489.50 ^i^	18.20 ^i^	713.00 ^i^	0.5750 ^i^	7.00451 ^u^	672.90 ^u^
*n*-eicosane	C_20_H_42_	112-95-8	282.56	767.00 ^i^	617.00 ^i^	11.10 ^i^	1190.00 ^h^	0.9070 ^i^	8.33954 ^t^	593.66 ^t^
*n*-heptane	C_7_H_16_	142-82-5	100.21	540.30 ^i^	371.60 ^i^	27.40 ^i^	432.00 ^i^	0.3490 ^i^	5.94356 ^u^	404.05 ^u^
n-hexadecane	C_16_H_34_	544-76-3	226.45	722.00 ^i^	560.00 ^i^	14.10 ^i^	930.00 ^i^	0.7420 ^i^	7.36480 ^u^	1669.19 ^u^
*n*-hexane	C_6_H_14_	110-54-3	86.18	507.50 ^i^	341.90 ^i^	30.10 ^i^	370.00 ^i^	0.2990 ^i^	5.61841 ^u^	434.76 ^u^
nitrous oxide	N_2_O	10024-97-2	44.01	309.60 ^i^	184.70 ^i^	72.40 ^i^	97.40 ^i^	0.1650 ^i^	3.67545 ^t^	239.63 ^t^
*n*-octane	C_8_H_18_	111-65-9	114.23	568.80 ^i^	398.80 ^i^	24.90 ^i^	492.00 ^i^	0.3980 ^i^	6.17328 ^u^	478.32 ^u^
*n*-propylbenzene	C_9_H_12_	103-65-1	120.20	638.20 ^i^	432.40 ^i^	32.00 ^i^	440.00 ^i^	0.3440 ^i^	5.99624 ^t^	493.97 ^t^
*n*-tetradecane	C_14_H_30_	629-59-4	198.39	693.00 ^i^	526.70 ^i^	14.40 ^i^	830.00 ^i^	0.5810 ^i^	7.68286 ^t^	536.38 ^t^
octafluorotoluene	C_7_F_8_	434-64-0	236.06	534.47 ^g^	377.73 ^g^	27.05 ^g^	428.00 ^g^	0.4758 ^d^	5.97931 ^t^	413.68 ^t^
*o*-difluorobenzene	C_6_H_4_F_2_	367-11-3	114.09	554.46 ^h^	364.66 ^h^	40.67 ^h^	299.50 ^h^	0.3200 ^h,b^	5.33270 ^t^	429.15 ^t^
oxygen	O_2_	7782-44-7	32.00	154.60 ^i^	90.20 ^i^	50.40 ^i^	73.40 ^i^	0.0250 ^i^	3.29728 ^t^	119.66 ^t^
*o*-xylene	C_8_H_10_	95-47-6	106.17	630.30 ^i^	417.60 ^i^	37.30 ^i^	369.00 ^i^	0.3100 ^i^	5.70029 ^t^	487.85 ^t^
palladium(II) acetylacetonate	C_10_H_14_O_4_Pd	14024-61-4	304.64	651.12 ^b^	573.15 ^n^	4.13 ^b^	435.41 ^b^	1.0014 ^d^	4.90200 ^x^	994.14 ^x^
*p*-chloronitrobenzene	C_6_H_4_ClNO_2_	100-00-5	157.56	751.00 ^h^	515.15 ^h^	39.80 ^h^	432.00 ^h^	0.4910 ^h^	5.89621 ^t^	581.27 ^t^
*p*-difluorobenzene	C_6_H_4_F_2_	540-36-3	114.09	556.00 ^h^	362.00 ^h^	44.00 ^h^	299.50 ^h^	0.2990 ^h^	5.20720 ^t^	430.34 ^t^
pentafluorobenzene	C_6_HF_5_	363-72-4	168.07	530.97 ^g^	358.89 ^g^	35.31 ^g^	324.00 ^g^	0.3711 ^d^	5.49825 ^t^	410.97 ^t^
phenanthrene	C_14_H_10_	85-01-8	178.23	873.00 ^i^	613.00 ^i^	29.00 ^h^	554.00 ^i^	0.4950 ^h^	6.77034 ^t^	675.70 ^t^
phenylbutazone	C_19_H_20_N_2_O_2_	50-33-9	308.38	861.18 ^e^	674.85 ^e^	18.38 ^e^	933.55 ^e^	1.0126 ^d^	7.63140 ^t^	666.55 ^t^
propane	C_3_H_8_	74-98-6	44.09	369.80 ^i^	231.10 ^i^	42.50 ^i^	203.00 ^i^	0.1530 ^i^	4.50412 ^u^	457.99 ^u^
propene	C_3_H_6_	115-07-1	42.08	364.90 ^i^	225.50 ^i^	46.00 ^i^	181.00 ^i^	0.1440 ^i^	4.49020 ^t^	282.43 ^t^
*p*-xylene	C_8_H_10_	106-42-3	106.17	616.20 ^i^	411.50 ^i^	35.10 ^i^	379.00 ^i^	0.3200 ^i^	5.76754 ^t^	476.94 ^t^
pyrene	C_16_H_10_	129-00-0	202.26	936.00 ^h^	667.95 ^h^	26.10 ^h^	630.00 ^h^	0.5090 ^h^	7.11077 ^t^	724.46 ^t^
quercetin	C_15_H_10_O_7_	117-39-5	302.24	1468.74 ^f^	1187.59 ^f^	66.64 ^f^	730.50 ^f^	2.4842 ^d^	6.17951 ^t^	1136.80 ^t^
squalene	C_30_H_50_	111-02-4	410.73	716.50 ^s^	678.39 ^q^	7.03 ^s^	1601.00 ^f^	0.6380 ^d^	9.46409 ^t^	554.57 ^t^
*s*-trioxane	C_3_H_6_O_3_	110-88-3	90.08	604.00 ^h^	387.65 ^h^	58.20 ^h^	206.00 ^h^	0.3340 ^h^	4.89292 ^t^	467.50 ^t^
sulfur hexafluoride	SF_6_	2551-62-4	146.05	318.70 ^i^	209.60 ^i^	37.60 ^i^	198.80 ^i^	0.2860 ^i^	4.76629 ^u^	271.68 ^u^
tetrabutyltin	C_16_H_36_Sn	1461-25-2	347.17	767.97 ^b^	548.45 ^c^	17.25 ^b^	760.75 ^b^	0.3212 ^d^	7.53290 ^t^	594.41 ^t^
tetraethyltin	C_8_H_20_Sn	597-64-8	234.95	655.92 ^b^	456.25 ^c^	25.75 ^b^	429.28 ^b^	0.3747 ^d^	6.45047 ^t^	507.68 ^t^
tetramethyltin	C_4_H_12_Sn	594-27-4	178.85	511.77 ^b^	347.65 ^c^	34.18 ^b^	263.54 ^b^	0.3807 ^d^	5.49115 ^t^	396.11 ^t^
tetrapropyltin	C_12_H_28_Sn	2176-98-9	291.06	759.88 ^b^	536.35 ^c^	20.66 ^b^	595.01 ^b^	0.3479 ^d^	7.16031 ^t^	588.15 ^t^
toluene	C_7_H_8_	108-88-3	92.14	591.80 ^i^	383.80 ^i^	41.00 ^i^	316.00 ^i^	0.2630 ^i^	5.45450 ^u^	350.74 ^u^
vitamin K_3_	C_11_H_8_O_2_	58-27-5	172.18	893.85 ^e^	638.20 ^f^	31.96 ^e^	537.20 ^e^	0.6105 ^d^	6.62867 ^t^	691.84 ^t^
water	H_2_ O	7732-18-5	18.02	647.30 ^i^	373.20 ^i^	221.20 ^i^	57.10 ^i^	0.3440 ^i^	3.24681 ^t^	501.01 ^t^
xenon	Xe	7440-63-3	131.30	289.70 ^i^	165.00 ^i^	58.40 ^i^	118.40 ^i^	0.0080 ^i^	3.85754 ^t^	224.23 ^t^

^a^ Taken from Valderrama and Rojas [53]; ^b^ Estimated by the Klincewicz method [31,48]; ^c^ Taken from ChemSpider [38]; ^d^ Estimated by the Lee-Kesler relation [54]; ^e^ Average of the values by the Joback [31,45,46] and Ambrose [31,49,50,51] methods; ^f^ Estimated by the Joback method [31,45,46]; ^g^ Taken from Korea Thermophysical Properties Data Bank (KDB) [39]; ^h^ Taken from Yaws (1998) [26]; ^i^ taken from Reid et al. [31]; ^j^ Taken from DIPPR database [40]; ^k^ Average of the values by the Joback [31,45,46] and Wen-Qiang [52] methods; ^l^ Taken from Yaws (2008) [41]; ^m^ Taken from sigma Aldrich data sheet; ^n^ Taken from LookChem [42]; ^o^ Taken from Zêzere et al. [56]; ^p^ Taken from Leite et al. [57]; ^q^ Taken from ASPEN database [43]; ^r^ Average of the values by the Joback [31,45,46] and Somayajulu [47] methods; ^s^ Taken from Catchpol et al. [58]; ^u^ Taken from Silva and Liu 2008 [59]; ^t^ Estimated by Equations (8) and (9) from reference [14]; ^v^ Taken from Cordeiro et al. [60]; *^x^* Taken from Cordeiro [44]; ^y^ Estimated by the Pitzer [55] equation.

**Table 3:** Required inputs for the new and classic diffusivity models.

Parameters	Proposed Models		Classic Models			
	ML Polar	ML Nonpolar	Wilke-Chang (Equation (1))	Tyn-Calus (Equation (3))	Magalhães et al. [9] (Equation (4))	Zhu et al. [13] (Equations (5)–(10))
T	●	●	●	●	●	●
$\rho_{1}$						●
$\mu_{1}$	●	●	●	●	●	
$M_{2}$	●	●				
$T_{c,2}$						●
$T_{\mathrm{bp},2}$						
$P_{c,2}$	●	●				●
$V_{c,2}$			●	●		
$w_{2}$						
$\sigma_{\mathrm{LJ},2}$						
$\varepsilon_{\mathrm{LJ},2}/k_{B}$						
$M_{1}$	●	●	●			●
$T_{c,1}$						●
$T_{\mathrm{bp},1}$						
$P_{c,1}$						
$V_{c,1}$				●		●
$w_{1}$						
$\sigma_{\mathrm{LJ},1}$						
$\varepsilon_{\mathrm{LJ},1}/k_{B}$	●					
Fitted	-	-	-	-	2	-
Count	6	5	4	4	4	7

Note: The ● indicates the parameters required in each model.

**Table 4:** Performance of several machine learning (ML)models for the prediction of diffusivities in polar systems (test set) and comparison with classic predictive and correlation models.

Model	NSys	NDP	Global AARD (%)	AARD_arith_ (%)	AARD_min_ (%)	AARD_max_ (%)	RMSE	*Q*^2^ (*R*^2^) ***
ML Polar Multilinear Regression	79	430	84.65	80.65	4.00	899.66	3.33 × 10^−5^	0.7215 (0.7504)
ML Polar *k*-Nearest Neighbors	79	430	8.94	17.55	0.22	317.43	1.20 × 10^−5^	0.9641 (1.0000)
ML Polar Decision Tree	79	430	7.14	12.68	0.22	229.69	7.83 × 10^−6^	0.9846 (1.0000)
ML Polar Random Forest	79	430	5.67	9.44	0.04	82.92	6.67 × 10^−6^	0.9889 (1.0000)
ML Polar Gradient Boosted	79	430	5.07	8.00	0.08	76.23	5.68 × 10^−6^	0.9919 (0.9998)
Wilke-Chang	79	430	40.92	41.35	1.37	197.71	3.15 × 10^−5^	0.7519 (0.6790)
Tyn-Calus	79	430	46.49	38.41	2.88	97.11	2.30 × 10^−5^	0.8672 (0.8399)
Magalhães et al.	76 *	419	5.19	6.23	0.15	92.77	5.81 × 10^−6^	0.9917 (0.9977)
Zhu et al.	**	**	**	**	**	**	**	**

* Magalhães et al. correlation cannot be applied in three systems of the database due to the low number of points. ** Model of Zhu et al. is not applicable to polar systems. NSys: number of systems; NDP: number of data points; Global AARD: weighted deviation of all systems; AARD_arith_: arithmetic average of all systems; AARD_min_: minimum AARD; and AARD_max_: maximum AARD. *** *Q*^2^ (*R*^2^): *R*^2^ is the coefficient of determination for training and *Q*^2^ is the corresponding value for testing, in the case of ML models. For the Wilke-Chang, Tyn-Calus and Zhu et al. models all values are predicted.

**Table 5:** Performance of several machine learning (ML) models for the prediction of diffusivities in nonpolar systems (test set) and comparison with classic predictive and correlation models.

Model	NSys	NDP	Global AARD (%)	AARD_arith_(%)	AARD_min_(%)	AARD_max_(%)	RMSE	*Q*^2^ (*R*^2^) **
ML Nonpolar Multilinear Regression	130	342	96.65	111.95	0.91	1731.52	8.37 × 10^−5^	0.5590 (0.5779)
ML Nonpolar *k*-Nearest Neighbors	130	342	13.64	13.86	0.00	63.05	2.93 × 10^−5^	0.9461 (0.9998)
ML Nonpolar Decision Tree	130	342	13.29	14.08	0.00	90.96	5.08 × 10^−5^	0.8380 (0.9998)
ML Nonpolar Random Forest	130	342	9.94	10.29	0.00	62.04	1.83 × 10^−5^	0.9789 (0.9998)
ML Nonpolar Gradient Boosted	130	342	5.86	6.02	0.03	25.87	1.39 × 10^−5^	0.9879 (0.9866)
Wilke-Chang	130	342	29.19	28.20	0.26	172.30	6.66 × 10^−5^	0.7214 (0.5546)
Tyn-Calus	130	342	28.84	27.82	0.18	64.97	7.01 × 10^−5^	0.6909 (0.7465)
Magalhães et al.	125 *	324	6.19	6.21	0.04	128.38	1.82 × 10^−5^	0.9801 (0.9890)
Zhu et al.	130	342	37.93	45.19	1.40	222.45	6.35 × 10^−5^	0.7466 (0.8343)

* Magalhães et al. correlation cannot be applied in five systems of the database due to the low number of points. NSys: number of systems; NDP: number of data points; Global AARD: weighted deviation of all systems; AARD_arith_: arithmetic average of all systems; AARD_min_: minimum AARD; and AARD_max_: maximum AARD. ** *Q*^2^ (*R*^2^): *R*^2^ is the coefficient of determination for training and *Q*^2^ is the corresponding value for testing, in the case of ML models. For the Wilke-Chang, Tyn-Calus and Zhu et al. models all values are predicted.

**Table 6:** Calculated deviations of the individual systems of the polar database (divided into test and train sets) achieved by the best machine learning model of this work (Gradient Boosted) and classic equations adopted for comparison.

		NDP			AARD (%)								Data Ref.
					ML Gradient Boosted		Wilke-Chang		Tyn-Calus		Magalhães et al.		
Solvent	Solute	Total	Test	Train	Test	Train	Test	Train	Test	Train	Test	Train	
1-propanol	ammonia	31	14	17	5.65	0.60	33.93	31.25	19.49	21.11	4.53	2.23	[71]
1-propanol	carbon dioxide	27	11	16	1.74	0.69	54.34	57.12	71.29	73.03	3.57	2.73	[71]
1-propanol	propane	36	9	27	4.04	0.87	48.26	53.11	62.76	66.25	4.84	4.87	[71]
1-propanol	propene	36	12	24	2.66	1.22	51.82	56.37	66.01	69.22	3.84	4.81	[71]
1-propanol	water	5	2	3	38.77	0.16	153.58	119.30	46.19	26.42	18.77	0.93	[72]
2-propanol	benzene	10	2	8	1.61	0.18	19.82	8.26	35.37	26.16	28.53	6.52	[73]
2-propanol	naphthalene	10	3	7	0.93	0.23	7.64	13.02	24.72	24.05	9.06	10.74	[73]
2-propanol	*n*-decane	10	3	7	0.74	0.20	11.68	20.45	23.09	30.72	3.81	15.80	[73]
2-propanol	*n*-tetradecane	9	5	4	6.36	0.72	15.44	14.88	20.85	21.60	24.45	2.49	[73]
2-propanol	phenanthrene	9	3	6	10.06	0.46	23.85	5.46	34.66	13.53	92.77	1.72	[73]
2-propanol	toluene	10	1	9	7.16	0.19	18.91	9.87	36.94	26.77	13.69	8.03	[73]
2-propanol	water	5	1	4	41.12	0.44	130.88	143.02	33.10	40.10	4.57	0.83	[72]
acetone	1,2,4-trichlorobenzene	6	2	4	5.85	0.48	10.53	11.95	27.10	28.26	3.59	1.08	[74]
acetone	1,3,5-trimethylbenzene	5	2	3	0.75	0.06	18.81	19.10	32.77	33.01	0.15	0.61	[74]
acetone	benzene	6		6		0.19		13.32		34.40		0.36	[74]
acetone	biphenyl	6	1	5	4.35	0.63	18.79	18.79	30.99	30.99	0.46	0.40	[74]
acetone	chlorobenzene	6		6		0.14		13.57		32.58		0.85	[74]
acetone	ethylbenzene	6	1	5	0.08	0.23	18.76	19.07	34.44	34.68	0.17	0.43	[74]
acetone	naphthalene	5		5		0.28		18.33		32.93		0.42	[74]
acetone	*n*-propylbenzene	5	4	1	0.98	0.00	21.09	21.14	34.47	34.52			[74]
acetone	toluene	5		5		0.12		16.89		34.87		0.38	[74]
acetone	water	4	1	3	5.94	0.06	83.53	85.64	6.60	7.82	0.80	0.87	[75]
acetonitrile	[Bmim][bti]	5	1	4	2.19	0.51	50.27	49.10	48.63	47.43	0.60	1.19	[76]
acetonitrile	[Emim][bti]	5	1	4	1.63	0.02	47.83	46.64	47.25	46.06	1.10	1.35	[76]
acetonitrile	[Hmim][bti]	5		5		0.29		48.77		46.06		1.94	[76]
acetonitrile	[Omim][bti]	5	1	4	1.12	0.26	48.99	49.22	45.36	45.61	0.18	1.04	[76]
acetonitrile	carbon disulfide	5	3	2	16.39	3.76	22.64	28.64	41.91	46.42	10.72		[77]
acetonitrile	methanol	20	6	14	6.49	0.96	20.28	15.79	43.25	40.05	1.44	1.78	[77]
chlorobenzene	propene	32	9	23	0.95	0.25	9.43	9.88	32.77	32.49	1.01	1.12	[78,79]
chlorotrifluoromethane	1,3-dibromobenzene	12	3	9	9.31	1.21	147.23	148.48	75.18	76.06	6.85	4.14	[80]
chlorotrifluoromethane	acetone	10	2	8	16.17	0.78	93.87	93.66	24.18	24.05	8.00	3.55	[80]
chlorotrifluoromethane	*p*-xylene	8	1	7	7.05	0.65	75.61	98.40	24.84	41.04	2.31	3.68	[80]
deuterium oxide	oxygen	18	7	11	5.43	0.27	20.33	16.57	38.87	35.99	4.64	7.55	[81]
ethanol	1,2-butanediol	5	2	3	37.20	1.27	30.65	27.41	13.27	15.42	2.61	0.24	[82]
ethanol	1,3,5-trimethylbenzene	13	5	8	4.09	0.54	13.22	18.95	21.13	19.42	1.65	1.92	[83]
ethanol	1,4-butanediol	4	4		63.79		48.40		2.88				[82]
ethanol	1-butanol	4	3	1	20.44	3.64	17.25	17.95	22.96	22.49			[82]
ethanol	2-phenylethyl acetate	15	4	11	2.64	1.38	16.89	17.80	38.86	39.53	2.98	1.97	[84]
ethanol	3-phenylpropyl acetate	15	3	12	2.59	0.91	14.30	13.49	35.82	35.21	3.93	1.76	[84]
ethanol	ammonia	18	5	13	3.84	2.00	36.24	42.92	29.11	25.63	5.32	3.18	[71]
ethanol	benzene	21	8	13	3.42	1.16	27.35	24.37	25.74	35.54	6.16	12.16	[82,83]
ethanol	benzonitrile	16	8	8	1.86	0.97	24.97	25.34	48.86	49.11	0.83	1.02	[85]
ethanol	benzyl acetate	15	5	10	4.43	0.98	17.97	13.93	41.27	38.38	3.36	2.89	[84]
ethanol	carbon dioxide	27	9	18	4.82	2.21	49.74	46.56	72.64	70.90	5.08	3.73	[71]
ethanol	chromium(III) acetylacetonate	9	1	8	7.17	0.77	20.79	16.81	8.31	11.33	2.99	2.24	[86,87]
ethanol	dibenzyl ether	15	5	10	3.00	1.52	22.47	25.90	41.47	44.06	4.26	1.37	[84]
ethanol	disperse blue 14	8	2	6	2.75	5.23	22.10	22.73	38.77	39.26	5.61	10.24	[88]
ethanol	disperse orange 11	12	2	10	0.44	0.17	20.42	15.17	38.89	34.86	6.15	2.75	[88]
ethanol	ethylene glycol	5	2	3	76.23	0.05	61.03	57.90	4.28	2.65	5.06	1.36	[82]
ethanol	eucalyptol	12	4	8	7.02	1.06	10.58	13.85	34.55	36.94	0.48	0.65	[56]
ethanol	gallic acid	24	5	19	5.14	0.61	134.06	132.71	53.92	53.04	1.57	0.79	[57]
ethanol	glycerol	5		5		1.08		52.51		4.59		3.28	[82]
ethanol	Ibuprofen	16	7	9	4.87	1.07	4.97	5.51	19.05	18.63	0.92	0.81	[89]
ethanol	naphthalene	13	2	11	8.86	0.16	21.43	14.25	30.88	20.36	11.33	1.13	[83]
ethanol	nitrous oxide	5		5		0.26		44.94		69.83		0.68	[90]
ethanol	palladium(II) acetylacetonate	4	1	3	4.84	0.03	15.52	18.85	17.74	15.36	0.67	0.80	[87]
ethanol	phenanthrene	13	2	11	11.23	0.06	4.25	11.34	22.56	17.30	2.83	1.26	[83]
ethanol	phenylbutazone	8	1	7	7.87	2.02	10.27	10.72	10.26	9.89	2.01	2.13	[91]
ethanol	propane	30	7	23	4.31	1.93	43.06	42.56	64.52	64.21	7.48	8.90	[71]
ethanol	propene	30	5	25	1.78	1.52	43.30	45.74	65.37	66.86	7.80	7.72	[71]
ethanol	quercetin	16	6	10	7.15	1.79	40.58	40.59	9.60	9.61	0.86	1.11	[92]
ethanol	toluene	14	7	7	5.02	0.12	20.45	17.54	24.14	20.86	8.93	0.70	[83]
ethanol	water	15	2	13	15.26	0.90	131.04	145.20	15.31	22.37	4.86	4.30	[75,82,93]
ethyl acetate	astaxanthin	12	5	7	1.50	0.56	11.44	14.29	8.83	11.61	1.51	2.85	[94]
ethyl acetate	quercetin	16	4	12	2.90	0.52	44.69	50.17	19.78	24.31	3.18	1.80	[92]
ethyl acetate	squalene	12	2	10	2.01	0.57	7.70	8.86	12.34	13.44	1.54	0.98	[94]
ethylene glycol	propene	31	9	22	1.36	0.86	48.94	48.81	64.24	64.14	1.41	1.70	[78,79]
methanol	[Bmim][bti]	11	5	6	3.46	1.69	42.58	41.65	54.56	53.82	5.00	2.15	[76,95]
methanol	[Emim][bti]	11	4	7	5.07	0.23	40.25	41.85	53.72	54.96	5.24	1.64	[76,95]
methanol	[Hmim][bti]	5	2	3	4.25	0.54	36.57	39.04	48.83	50.82	3.91	0.74	[76]
methanol	[Omim][bti]	5		5		0.61		39.02		49.96		1.33	[76]
methanol	1,3,5-trimethylbenzene	4		4		1.15		15.85		42.38		3.25	[73]
methanol	acetonitrile	26	9	17	2.94	1.30	27.88	26.50	57.94	57.13	2.19	1.63	[77]
methanol	ammonia	24	6	18	0.93	1.78	106.11	114.44	3.67	7.78	4.25	3.93	[71]
methanol	benzene	4	1	3	2.79	0.55	1.88	12.49	38.59	45.23	3.28	4.28	[73]
methanol	carbon dioxide	25	10	15	3.80	0.79	30.72	30.86	63.70	63.77	4.05	3.84	[71]
methanol	carbon monoxide	8	1	7	4.88	0.52	23.36	14.78	59.89	55.02	8.75	3.60	[96]
methanol	disperse blue 14	8	2	6	3.70	0.59	57.69	51.97	67.99	63.66	8.22	1.11	[88]
methanol	disperse orange 11	16	5	11	2.65	0.26	51.25	52.71	63.97	65.05	3.01	1.96	[88]
methanol	naphthalene	4	2	2	7.59	0.08	17.60	15.98	44.04	42.94	19.90		[73]
methanol	*p*-chloronitrobenzene	18	7	11	1.47	0.60	22.46	22.66	46.93	47.06	1.08	1.07	[97]
methanol	phenanthrene	4	1	3	13.71	0.48	12.15	21.29	37.19	43.73	4.44	3.14	[73]
methanol	propane	27	11	16	2.14	1.50	24.08	27.00	54.47	56.22	2.43	2.45	[71]
methanol	toluene	4		4		0.25		14.12		44.35		3.66	[73]
methanol	vitamin K_3_	4		4		0.45		25.59		47.09		0.45	[98]
methanol	water	5	2	3	28.86	0.74	310.36	281.35	97.11	83.18	11.09	0.18	[99]
n-butanol	ammonia	64	17	47	2.63	1.75	38.12	38.41	20.73	20.56	5.36	5.81	[71]
n-butanol	carbon dioxide	66	19	47	1.19	1.06	47.26	45.51	68.33	67.28	5.98	6.27	[71]
n-butanol	propane	98	33	65	1.86	1.51	49.70	49.52	65.43	65.31	2.58	3.15	[71]
n-butanol	propene	135	45	90	2.83	1.66	50.48	48.53	66.64	65.33	5.15	3.90	[71]

**Table 7:** Calculated deviations of the individual systems of the nonpolar database (divided into test and train sets) achieved by the best machine learning model of this work (Gradient Boosted) and classic equations adopted for comparison.

Solvent	Solute	NDP			AARD (%)										Data Ref.
					ML Gradient Boosted		Wilke-Chang		Tyn-Calus		Magalhães et al.		Zhu et al.		
		Total	Test	Train	Test	Train	Test	Train	Test	Train	Test	Train	Test	Train	
2,2,4-trimethylpentane	1,3,5-trimethylbenzene	4		4		2.11		21.44		17.70		0.64		171.90	[100]
2,2,4-trimethylpentane	benzene	4	1	3	2.49	1.36	11.31	14.98	31.05	28.78	0.04	2.33	128.60	119.69	[100]
2,2,4-trimethylpentane	ethylbenzene	4		4		3.68		19.42		21.11		1.79		157.43	[100]
2,2,4-trimethylpentane	*o*-xylene	4		4		1.96		16.19		23.43		2.78		147.48	[100]
2,2,4-trimethylpentane	*p*-xylene	4	1	3	6.04	6.76	15.57	5.11	23.48	33.84	4.27	2.74	116.04	126.93	[100]
2,2,4-trimethylpentane	toluene	4		4		2.21		10.10		29.38		2.07		126.50	[100]
2,3-dimethylbutane	benzene	11	2	9	3.22	3.10	14.74	13.29	40.85	39.84	1.78	1.74	9.45	7.59	[101]
2,3-dimethylbutane	naphthalene	9	2	7	1.28	1.68	18.35	19.02	38.53	39.04	0.61	2.18	1.80	2.59	[101]
2,3-dimethylbutane	phenanthrene	11	2	9	0.65	0.63	20.75	20.51	37.19	36.99	2.44	1.63	2.39	5.87	[101]
2,3-dimethylbutane	toluene	10	2	8	2.52	3.36	15.89	17.53	39.58	40.75	2.84	2.17	4.97	4.77	[101]
cyclohexane	1,1′-dimethylferrocene	5	2	3	1.07	1.64	9.73	8.30	17.40	18.48	2.41	0.26	192.52	197.96	[102]
cyclohexane	1,3,5-trimethylbenzene	12	1	11	9.04	3.82	6.73	14.13	28.83	14.33	8.28	8.32	16.07	59.79	[103,104]
cyclohexane	acetone	4	2	2	2.31	0.01	20.96	19.77	46.91	46.10	0.96		106.99	92.31	[104]
cyclohexane	argon	7	3	4	9.78	4.63	6.89	2.54	43.32	44.85	5.54	2.05	40.33	66.48	[105]
cyclohexane	benzene	12	2	10	12.00	2.96	24.55	17.57	13.13	18.78	12.40	8.05	92.05	61.13	[104,106]
cyclohexane	carbon tetrachloride	7	2	5	0.50	1.02	15.04	23.35	18.88	13.02	3.28	0.96	53.23	103.63	[105]
cyclohexane	ethane	5	1	4	13.53	1.23	3.43	2.22	34.57	37.18	0.29	1.23	183.33	86.88	[107]
cyclohexane	ethylene	5	1	4	1.93	1.06	0.26	1.74	37.99	37.80	1.60	1.08	66.73	110.83	[107]
cyclohexane	ethylferrocene	6	1	5	0.68	0.49	5.53	8.18	20.56	18.56	1.18	0.75	178.04	169.05	[102]
cyclohexane	ferrocene	5	3	2	2.84	0.08	15.24	13.62	16.70	17.87	1.37	0.20	49.79	58.60	[102]
cyclohexane	krypton	6	3	3	9.01	2.60	16.32	15.16	32.42	33.09	3.07	1.27	54.85	78.43	[105]
cyclohexane	methane	6	4	2	13.80	0.41	9.74	9.08	46.78	46.39	7.63		49.30	22.59	[105]
cyclohexane	m-xylene	4		4		1.01		21.96		41.90		1.29		94.56	[104]
cyclohexane	naphthalene	12	4	8	10.33	3.64	14.64	10.87	14.91	18.18	9.98	6.90	41.94	39.98	[104,106]
cyclohexane	phenanthrene	8	3	5	5.64	1.43	4.82	4.27	19.02	23.03	4.82	2.49	4.34	7.53	[106]
cyclohexane	*p*-xylene	8		8		2.31		4.13		28.00		3.63		28.67	[106]
cyclohexane	tetrabutyltin	7	2	5	10.03	1.38	20.87	25.56	7.51	9.58	3.79	1.64	11.64	14.39	[105]
cyclohexane	tetraethyltin	7	2	5	0.61	1.78	24.37	24.29	7.91	8.24	1.43	2.17	57.43	57.01	[105]
cyclohexane	tetramethyltin	7	2	5	4.77	0.48	29.90	33.82	9.13	7.47	2.31	1.06	90.37	95.59	[105]
cyclohexane	tetrapropyltin	6	4	2	3.96	1.01	21.89	30.99	7.23	8.08	2.03		21.99	21.49	[105]
cyclohexane	toluene	12	2	10	8.49	3.06	16.22	10.84	18.75	20.21	11.65	7.31	51.76	56.57	[104,106]
cyclohexane	xenon	7	6	1	5.42	0.02	25.17	14.32	23.88	30.48			83.09	150.96	[105]
ethane	1-octene	6	2	4	6.96	1.27	3.27	5.54	1.86	5.43	5.88	1.96	17.16	9.15	[108]
ethane	1-tetradecene	9	4	5	6.06	0.28	20.10	20.84	13.67	14.48	3.84	3.71	21.78	13.41	[108]
*n*-decane	12-crown-4	4	1	3	8.73	4.44	20.77	23.64	17.52	15.56	0.66	4.99	42.67	40.09	[109]
*n*-decane	15-crown-5	4	1	3	8.77	1.24	41.39	22.03	0.18	13.54	22.17	0.69	28.31	21.11	[109]
*n*-decane	18-crown-6	4	1	3	2.17	2.05	30.58	25.53	4.63	8.31	14.50	4.29	3.54	4.36	[109]
*n*-decane	argon	3	2	1	11.89	0.11	12.79	10.56	44.32	55.28			26.35	91.32	[110]
*n*-decane	carbon tetrachloride	3		3		5.74		17.09		26.45		1.24		71.87	[110]
*n*-decane	dicyclohexano-18-crown-6	4		4		0.68		25.60		2.44		1.27		83.59	[109]
*n*-decane	dicyclohexano-24-crown-8	4	3	1	7.95	0.13	25.82	33.28	3.15	8.46			119.04	192.36	[109]
*n*-decane	krypton	3		3		2.49		23.85		35.90		3.27		69.93	[110]
*n*-decane	*s*-trioxane	4		4		2.24		24.60		25.63		0.91		50.71	[109]
*n*-decane	tetrabutyltin	4	1	3	3.53	0.71	29.41	29.09	2.91	3.22	1.57	0.96	22.54	20.34	[110]
*n*-decane	tetraethyltin	4	1	3	25.87	24.86	1.63	6.66	33.23	30.98	0.59	1.98	19.15	13.44	[110]
*n*-decane	tetramethyltin	4	2	2	4.38	7.90	37.59	36.56	14.25	14.90	2.61		75.10	68.10	[110]
*n*-decane	tetrapropyltin	4	1	3	0.83	1.65	26.81	29.97	8.87	6.60	0.68	2.00	26.72	24.34	[110]
*n*-decane	xenon	8	1	7	15.12	2.57	1.46	18.76	46.61	35.66	5.99	3.19	137.71	82.50	[110,111]
*n*-dodecane	1,3,5-trimethylbenzene	4	2	2	4.64	0.31	6.99	1.70	39.23	35.47	3.02		130.38	107.10	[104]
*n*-dodecane	acetone	5	1	4	6.18	0.82	5.13	4.64	45.44	45.15	0.90	1.37	98.06	103.24	[104]
*n*-dodecane	benzene	4	2	2	3.42	0.69	4.97	3.88	43.25	42.60	1.57	0.00	122.78	121.55	[104]
*n*-dodecane	carbon dioxide	9	3	6	5.85	2.86	61.83	88.25	19.08	9.16	11.39	1.56	30.14	22.67	[112]
*n*-dodecane	carbon monoxide	9	3	6	15.15	2.87	73.13	52.06	13.55	24.07	7.28	7.69	24.57	29.78	[112]
*n*-dodecane	hydrogen	9	5	4	7.78	6.84	25.13	21.17	64.97	63.11	10.12	9.67	47.72	49.66	[112]
*n*-dodecane	linoleic acid methyl ester	4		4		1.10		13.54		13.08		0.37		42.50	[104]
*n*-dodecane	*m*-xylene	4		4		1.39		10.17		42.74		0.62		108.09	[104]
*n*-dodecane	naphthalene	5	2	3	4.99	0.75	5.64	10.11	38.86	41.75	3.55	0.93	79.40	81.82	[104]
*n*-dodecane	*n*-decane	5	1	4	0.03	2.21	56.61	45.00	8.43	4.59	1.98	3.71	11.30	34.63	[113]
*n*-dodecane	*n*-hexadecane	5	1	4	10.77	1.08	65.59	57.28	23.68	17.47	5.92	0.83	19.21	19.32	[113]
*n*-dodecane	*n*-octane	9	6	3	2.31	0.16	47.87	50.94	6.18	3.73	10.34	1.17	33.86	8.63	[113]
*n*-dodecane	*n*-tetradecane	5	1	4	2.94	1.23	39.89	59.70	2.42	16.92	16.34	1.53	20.35	16.40	[113]
*n*-dodecane	toluene	4	2	2	5.84	0.79	7.90	11.57	43.05	45.33	2.72		95.30	125.51	[104]
*n*-dodecane	vitamin K_3_	4	1	3	0.19	0.22	10.31	11.59	39.14	40.01	0.22	0.98	34.63	38.39	[104]
*n*-eicosane	carbon dioxide	5	2	3	16.17	0.01	172.30	147.95	21.79	12.93	0.71	1.13	8.06	29.92	[114]
*n*-eicosane	carbon monoxide	5	2	3	10.88	4.29	114.69	136.89	8.76	7.81	0.55	0.54	50.15	19.59	[114]
*n*-eicosane	hydrogen	5	1	4	3.25	110.78	8.54	252.32	61.72	129.43	128.38	73.30	4.12	99.16	[114]
*n*-eicosane	*n*-dodecane	5	2	3	13.72	1.97	138.63	134.49	52.19	49.55	1.74	0.91	67.82	55.95	[114]
*n*-eicosane	n-hexadecane	5	4	1	16.16	1.99	141.26	144.68	61.19	63.48			61.96	30.75	[114]
*n*-eicosane	*n*-octane	5	2	3	7.53	0.63	134.16	124.83	39.95	34.37	2.54	1.67	54.76	57.07	[114]
*n*-heptane	1,3,5-trimethylbenzene	4	2	2	0.87	0.75	4.11	5.33	23.55	22.65	1.43		7.92	9.31	[115]
*n*-heptane	2,2,4-trimethylpentane	4	2	2	4.54	0.52	1.39	2.85	24.53	23.44	0.58	0.10	23.40	21.87	[116]
*n*-heptane	benzene	11	4	7	3.62	2.15	4.50	6.14	29.86	28.76	1.91	3.07	8.71	12.97	[115,117]
*n*-heptane	ethylbenzene	4		4		5.10		8.27		22.51		0.23		14.85	[115]
*n*-heptane	*n*-decane	6	1	5	4.29	2.79	15.13	6.99	33.96	24.47	8.69	2.42	10.94	5.52	[113,118]
*n*-heptane	*n*-dodecane	6	3	3	5.28	0.14	4.41	14.00	19.20	31.09	59.49	2.21	6.13	24.60	[113,118]
*n*-heptane	*n*-hexadecane	9	3	6	6.51	0.65	5.88	5.39	17.14	16.55	1.00	1.38	26.09	25.64	[119,120,121]
*n*-heptane	*n*-hexane	11	3	8	5.03	0.79	8.45	10.02	34.59	35.72	2.67	0.77	16.44	10.75	[113,119,121]
*n*-heptane	*n*-octane	13	3	10	7.32	1.72	7.36	9.29	27.53	31.88	2.94	1.28	4.50	2.94	[113,118]
*n*-heptane	*n*-tetradecane	6	3	3	2.73	1.12	7.65	9.45	21.46	22.01	2.56	1.51	28.62	33.71	[113,118]
*n*-heptane	*o*-xylene	4	2	2	4.35	2.02	7.08	2.49	29.03	29.16	0.62		3.70	3.50	[115]
*n*-heptane	*p*-xylene	4	1	3	6.86	2.70	5.35	7.22	32.10	33.44	0.70	0.66	1.40	1.36	[115]
*n*-heptane	toluene	4	3	1	5.71	1.04	3.63	5.00	33.00	27.03			4.30	5.86	[115]
*n*-hexadecane	carbon dioxide	10	4	6	2.02	1.81	92.63	112.99	13.75	15.19	7.11	4.53	37.48	32.03	[112]
*n*-hexadecane	carbon monoxide	10	3	7	3.49	3.59	80.63	91.32	16.76	13.82	3.77	4.83	52.93	49.96	[112]
*n*-hexadecane	hydrogen	10	7	3	6.89	1.04	24.77	18.43	59.19	54.00	12.88	0.99	38.66	34.17	[112]
*n*-hexadecane	*n*-decane	5	1	4	10.89	1.40	62.63	79.23	5.76	16.55	5.16	1.59	152.48	39.94	[122]
*n*-hexadecane	*n*-dodecane	5		5		1.16		75.79		17.71		2.72		55.76	[122]
*n*-hexadecane	*n*-octane	10	1	9	6.82	0.57	88.97	76.42	18.59	10.71	1.45	3.00	22.13	68.70	[122]
*n*-hexadecane	*n*-tetradecane	5	2	3	1.59	1.09	70.97	78.33	17.57	22.63	2.13	2.39	50.42	36.41	[122]
*n*-hexane	1,1′-dimethylferrocene	4	1	3	0.96	0.27	15.92	16.74	13.20	12.58	1.01	0.08	45.28	46.51	[102]
*n*-hexane	1,2,3,5-tetrafluorobenzene	7	2	5	4.31	3.24	20.21	17.52	41.62	39.64	1.22	5.13	7.14	10.04	[123]
*n*-hexane	1,2,4,5-tetrafluorobenzene	7	2	5	1.98	1.25	20.44	16.25	41.78	38.72	3.09	4.44	13.93	16.22	[123]
*n*-hexane	1,2,4-trifluorobenzene	7	2	5	4.61	0.87	24.28	14.88	45.04	38.22	5.76	1.46	12.96	6.40	[123]
*n*-hexane	1,3,5-trimethylbenzene	20	7	13	2.98	1.59	10.09	8.34	31.66	30.42	5.24	5.79	8.52	4.45	[103,104]
*n*-hexane	9,10-dimethylanthracene	8	4	4	13.89	3.00	12.79	19.02	27.56	32.73	6.22	0.32	116.02	83.34	[124]
*n*-hexane	acetone	5	2	3	2.70	1.08	5.05	3.73	36.55	34.67	5.60	1.05	10.75	4.24	[104]
*n*-hexane	acetonitrile	7		7		2.40		5.79		39.09		2.70		22.16	[125]
*n*-hexane	benzene	48	18	30	3.48	2.39	6.16	7.86	31.07	31.34	9.04	6.60	15.66	25.65	[103,104,107,123,125,126,127,128]
*n*-hexane	carbon disulfide	10	4	6	4.49	3.58	2.32	10.16	35.24	29.75	7.20	3.52	44.88	76.81	[125]
*n*-hexane	carbon tetrabromide	8	1	7	7.97	1.02	30.34	19.55	5.72	16.14	1.95	8.28	168.32	115.24	[124]
*n*-hexane	ethylferrocene	4		4		0.61		18.11		11.55		0.12		35.49	[102]
*n*-hexane	ferrocene	4	1	3	3.84	0.40	31.11	22.97	5.72	11.57	0.41	0.15	17.28	16.84	[123]
*n*-hexane	hexafluorobenzene	7	2	5	2.23	1.89	7.46	10.50	31.30	34.96	2.19	3.76	15.66	21.90	[123]
*n*-hexane	indole	2		2		0.62		10.64		32.22				13.24	[104]
*n*-hexane	linoleic acid methyl ester	2		2		2.02		2.08		12.90				95.99	[104]
*n*-hexane	*m*-xylene	5	2	3	1.77	0.04	9.32	8.01	32.84	31.87	1.82	2.56	5.26	4.57	[104]
*n*-hexane	naphthalene	21	5	16	3.43	2.44	12.23	11.95	33.92	33.71	4.95	4.32	8.19	10.88	[103,104,125,126]
*n*-hexane	*n*-heptane	11	5	6	4.88	1.16	13.00	12.35	29.03	33.49	7.30	0.93	13.25	2.53	[119,120,121,129]
*n*-hexane	*n*-octane	7	2	5	2.01	1.14	12.76	12.64	32.28	32.19	1.05	0.30	2.05	1.68	[119,129]
*n*-hexane	octafluorotoluene	7	1	6	0.23	0.26	21.39	8.53	40.45	30.16	4.30	2.92	13.40	15.97	[123]
*n*-hexane	*o*-difluorobenzene	7	2	5	2.25	0.86	9.72	12.64	35.75	37.83	4.29	2.44	3.57	16.35	[123]
*n*-hexane	*p*-difluorobenzene	7	3	4	2.62	0.44	19.67	9.73	42.83	35.76	24.35	0.79	27.69	2.93	[123]
*n*-hexane	pentafluorobenzene	7	1	6	2.79	0.39	1.84	12.11	26.52	36.58	1.96	3.78	6.06	17.91	[123]
*n*-hexane	phenanthrene	15	6	9	3.33	1.60	14.07	14.25	31.89	32.04	4.37	5.72	14.18	11.93	[103]
*n*-hexane	*p*-xylene	17	4	13	6.35	2.44	15.89	10.62	37.62	33.72	4.15	4.56	9.32	8.04	[103,104]
*n*-hexane	pyrene	8	2	6	10.54	10.51	62.03	50.27	31.35	21.81	8.62	4.72	153.51	103.03	[124,126]
*n*-hexane	toluene	32	14	18	4.58	2.65	8.46	8.14	32.19	30.74	4.98	3.72	12.56	19.33	[103,104,130,131]
*n*-hexane	vitamin K_3_	5	1	4	3.32	1.01	11.31	16.36	30.09	34.07	0.78	0.73	34.19	37.24	[104,132]
*n*-octane	1,3,5-trimethylbenzene	8	3	5	2.31	1.74	7.21	6.99	23.51	23.67	0.47	0.62	23.97	22.64	[103,104]
*n*-octane	argon	4	1	3	1.76	5.82	6.69	14.09	44.01	41.09	1.93	1.30	23.81	18.74	[110]
*n*-octane	benzene	8	2	6	1.90	0.79	2.80	2.87	34.64	35.64	0.29	0.20	15.45	17.40	[100,115]
*n*-octane	carbon tetrachloride	4		4		1.24		15.76		23.67		1.05		34.90	[110]
*n*-octane	ethylbenzene	8	4	4	6.65	6.50	3.65	7.21	28.14	25.44	3.22	1.19	24.94	22.80	[100,115]
*n*-octane	krypton	4	1	3	14.06	1.75	22.31	30.11	33.56	29.32	3.72	0.39	40.36	36.12	[110]
*n*-octane	methane	4	1	3	9.77	2.46	10.46	3.34	50.64	45.68	6.32	0.40	45.79	10.22	[110]
*n*-octane	*n*-heptane	7	4	3	6.34	0.55	11.43	11.60	36.84	36.96	1.42	0.12	20.87	17.70	[119,133]
*n*-octane	*n*-hexane	6	4	2	4.66	0.29	5.47	6.95	34.39	35.42	3.46		34.50	22.19	[119]
*n*-octane	*o*-xylene	8		8		1.39		1.30		31.53		0.73		14.39	[100,115]
*n*-octane	*p*-xylene	8	1	7	5.49	3.85	9.50	8.80	36.92	36.43	0.99	0.83	4.21	7.47	[100,115]
*n*-octane	tetrabutyltin	4	1	3	0.14	1.85	21.42	33.04	4.40	10.56	14.34	3.93	5.22	14.77	[110]
*n*-octane	tetraethyltin	5		5		4.29		34.09		14.16		3.79		17.89	[110]
*n*-octane	tetramethyltin	4		4		1.78		44.76		15.82		8.09		35.98	[110]
*n*-octane	tetrapropyltin	4	1	3	2.18	0.32	22.11	35.53	7.90	12.77	0.10	10.73	6.73	6.87	[110]
*n*-octane	toluene	8	1	7	0.28	0.53	1.25	3.08	31.64	33.55	1.92	1.28	12.43	17.20	[100,115]
*n*-octane	xenon	8	3	5	7.17	2.42	14.65	18.84	34.81	33.03	3.50	5.95	43.59	48.17	[110,111]
*n*-tetradecane	acridine	8	4	4	6.32	0.99	25.62	19.86	18.67	21.21	5.12	7.85	50.28	48.90	[134]
*n*-tetradecane	argon	4	1	3	13.85	3.81	4.21	24.13	55.48	60.17	3.71	4.35	44.66	76.49	[110]
*n*-tetradecane	benzothiophene	7	3	4	9.79	2.27	37.15	40.83	15.35	13.08	2.67	3.25	112.41	81.45	[134]
*n*-tetradecane	carbon tetrachloride	4		4		2.36		16.38		32.05		2.54		181.74	[110]
*n*-tetradecane	dibenzothiophene	8	3	5	12.20	2.93	31.28	40.52	14.59	8.58	7.34	2.43	58.10	73.29	[134]
*n*-tetradecane	krypton	4		4		4.58		17.50		49.11		6.70		102.06	[110]
*n*-tetradecane	methane	4	2	2	17.29	1.80	17.88	41.86	59.61	71.62	58.01		34.84	92.68	[110]
*n*-tetradecane	naphthalene	7		7		2.83		14.51		28.99		2.67		74.98	[134]
*n*-tetradecane	tetrabutyltin	4	2	2	17.37	1.95	40.27	36.01	11.38	5.16	4.94		116.75	115.45	[110]
*n*-tetradecane	tetraethyltin	4		4		3.09		29.87		18.07		5.56		143.59	[110]
*n*-tetradecane	tetramethyltin	4	2	2	13.08	0.06	29.05	40.68	25.25	18.52	6.64		202.70	152.93	[110]
*n*-tetradecane	tetrapropyltin	4	1	3	13.99	0.21	53.61	25.95	2.60	15.87	6.51	1.69	67.58	126.86	[110]
*n*-tetradecane	xenon	8	1	7	0.64	2.42	7.11	16.22	53.23	47.68	1.98	5.78	222.45	179.79	[110,111]
propane	1-octene	8	1	7	0.07	0.88	18.41	19.52	27.34	28.33	0.06	1.68	7.42	9.54	[108]
propane	1-tetradecene	8	3	5	3.54	0.38	36.40	30.97	36.84	31.45	3.52	0.98	48.48	31.59	[108]
sulfur hexafluoride	1,3,5-trimethylbenzene	10		10		0.86		90.68		28.87		4.43		14.17	[80]
sulfur hexafluoride	benzene	9	2	7	1.08	3.65	85.93	86.27	14.85	17.62	10.25	6.77	5.62	7.82	[80]
sulfur hexafluoride	benzoic acid	6	3	3	22.48	4.26	150.51	144.36	62.38	58.39	3.11	0.11	22.70	11.88	[135]
sulfur hexafluoride	carbon tetrachloride	7	2	5	2.81	1.69	95.35	134.58	22.01	46.52	2.71	1.86	33.23	12.98	[80]
sulfur hexafluoride	naphthalene	5	2	3	4.51	1.54	62.53	74.74	8.94	17.12	9.70	0.38	16.10	7.74	[135]
sulfur hexafluoride	*p*-xylene	52	14	38	4.09	2.16	88.28	88.44	24.32	24.42	2.51	4.62	5.61	8.54	[80]
sulfur hexafluoride	toluene	11	4	7	4.37	1.95	88.43	83.35	20.52	17.27	4.95	3.50	4.66	8.66	[80]

## Data Availability

No new data were created or analyzed in this study. Data sharing is not applicable to this article.

## Supplementary Materials

The following are available online at https://www.mdpi.com/1996-1944/14/3/542/s1, Software, Table S1: Tested and best hyper-parameter values for each machine learning algorithm, Figure S1: Predicted versus experimental diffusivities for the test set of polar systems using the Multilinear Regression model, Figure S2: Predicted versus experimental diffusivities for the test set of polar systems using the k-Nearest Neighbors model, Figure S3: Predicted versus experimental diffusivities for the test set of polar systems using the Decision Tree model, Figure S4: Predicted versus experimental diffusivities for the test set of polar systems using the Random Forest model, Figure S5: Predicted versus experimental diffusivities for the test set of nonpolar systems using the Multilinear Regression model, Figure S6: Predicted versus experimental diffusivities for the test set of nonpolar systems using the k-Nearest Neighbors model, Figure S7: Predicted versus experimental diffusivities for the test set of nonpolar systems using the Decision Tree model, Figure S8: Predicted versus experimental diffusivities for the test set of nonpolar systems using the Random Forest model, Figure S9: y-Randomization calculations for the selected ML Gradient Boosted models for (a) polar systems and (b) nonpolar systems. The bars show the *Q*2 values for models based on randomized diffusivity data. The dashed horizontal lines show the *Q*2 values of the actual models. Figure S10: Calculated versus experimental diffusivities for the test set of polar systems for the Tyn-Calus model. (a) full D12 range; (b) zoomed on lower D12 range, Figure S11: Calculated versus experimental diffusivities for the test set of nonpolar systems for the Tyn-Calus model. (a) full D12 range; (b) zoomed on lower D12 range, Figure S12: Calculated versus experimental diffusivities for the test set of nonpolar systems for the Zhu et al. model. (a) full D12 range; (b) zoomed on lower D12 range.

## References

[1] Wankat P.C. (1994). Rate-Controlled Separations.

[2] Oliveira E.L.G., Silvestre A.J.D., Silva C.M. (2011). Review of kinetic models for supercritical fluid extraction. Chem. Eng. Res. Des..

[3] Carberry J.J. (1971). Chemical and Catalytic Reaction Engineering.

[4] Zêzere B., Portugal I., Gomes J.R.B., Silva C.M. (2021). Revisiting Tracer Liu-Silva-Macedo model for binary diffusion coefficient using the largest database of liquid and supercritical systems. J. Supercrit. Fluids.

[5] Wilke C.R., Chang P. (1955). Correlation of diffusion coefficients in dilute solutions. AIChE J..

[6] Scheibel E.G. (1954). Liquid Diffusivities. Ind. Eng. Chem..

[7] Tyn M.T., Calus W.F. (1975). Diffusion Coefficients in Dilute Binary Liquid Mixtures. J. Chem. Eng. Data.

[8] Hayduk W., Minhas B.S. (1982). Correlations for prediction of molecular diffusivities in liquids. Can. J. Chem. Eng..

[9] Magalhães A.L., Lito P.F., Da Silva F.A., Silva C.M. (2013). Simple and accurate correlations for diffusion coefficients of solutes in liquids and supercritical fluids over wide ranges of temperature and density. J. Supercrit. Fluids.

[10] Magalhães A.L., Da Silva F.A., Silva C.M. (2012). Tracer diffusion coefficients of polar systems. Chem. Eng. Sci..

[11] Dymond J.H. (1974). Corrected Enskog theory and the transport coefficients of liquids. J. Chem. Phys..

[12] Silva C.M., Liu H. (2008). Modelling of Transport Properties of Hard Sphere Fluids and Related Systems, and its Applications. Theory and Simulation of Hard-Sphere Fluids and Related Systems.

[13] Zhu Y., Lu X., Zhou J., Wang Y., Shi J. (2002). Prediction of diffusion coefficients for gas, liquid and supercritical fluid: Application to pure real fluids and infinite dilute binary solutions based on the simulation of Lennard–Jones fluid. Fluid Phase Equilib..

[14] Magalhães A.L., Cardoso S.P., Figueiredo B.R., Da Silva F.A., Silva C.M. (2010). Revisiting the liu-silva-macedo model for tracer diffusion coefficients of supercritical, liquid, and gaseous systems. Ind. Eng. Chem. Res..

[15] Liu H., Silva C.M., Macedo E.A. (1997). New Equations for Tracer Diffusion Coefficients of Solutes in Supercritical and Liquid Solvents Based on the Lennard-Jones Fluid Model. Ind. Eng. Chem. Res..

[16] Gharagheizi F., Sattari M. (2009). Estimation of molecular diffusivity of pure chemicals in water: A quantitative structure-property relationship study. SAR QSAR Environ. Res..

[17] Khajeh A., Rasaei M.R. (2011). Diffusion coefficient prediction of acids in water at infinite dilution by QSPR method. Struct. Chem..

[18] Beigzadeh R., Rahimi M., Shabanian S.R. (2012). Developing a feed forward neural network multilayer model for prediction of binary diffusion coefficient in liquids. Fluid Phase Equilib..

[19] Eslamloueyan R., Khademi M.H. (2010). A neural network-based method for estimation of binary gas diffusivity. Chemom. Intell. Lab. Syst..

[20] Abbasi A., Eslamloueyan R. (2014). Determination of binary diffusion coefficients of hydrocarbon mixtures using MLP and ANFIS networks based on QSPR method. Chemom. Intell. Lab. Syst..

[21] Mirkhani S.A., Gharagheizi F., Sattari M. (2012). A QSPR model for prediction of diffusion coefficient of non-electrolyte organic compounds in air at ambient condition. Chemosphere.

[22] Rahimi M.R., Karimi H., Yousefi F. (2012). Prediction of carbon dioxide diffusivity in biodegradable polymers using diffusion neural network. Heat Mass Transf. Stoffuebertragung.

[23] Lashkarbolooki M., Hezave A.Z., Bayat M. (2017). Thermal diffusivity of hydrocarbons and aromatics: Artificial neural network predicting model. J. Thermophys. Heat Transf..

[24] Chudzik S. (2012). Measurement of thermal diffusivity of insulating material using an artificial neural network. Meas. Sci. Technol..

[25] Aniceto J.P.S., Zêzere B., Silva C.M. (2021). Machine learning models for the prediction of diffusivities in supercritical CO_2_ systems. J. Mol. Liq..

[26] Yaws C.L. (1998). Chemical Properties Handbook: Physical, Thermodynamic, Environmental, Transport, Safety, and Health Related Properties for Organic and Inorganic Chemicals.

[27] Cibulka I., Ziková M. (1994). Liquid densities at elevated pressures of 1-alkanols from C1 to C10: A critical evaluation of experimental data. J. Chem. Eng. Data.

[28] Cibulka I., Hnědkovský L., Takagi T. (1997). P−$ρ$−T data of liquids: Summarization and evaluation. 4. Higher 1-alkanols (C11, C12, C14, C16), secondary, tertiary, and branched alkanols, cycloalkanols, alkanediols, alkanetriols, ether alkanols, and aromatic hydroxy derivatives. J. Chem. Eng. Data.

[29] Cibulka I., Takagi T., Růžička K. (2000). P−ρ−T data of liquids: Summarization and evaluation. 7. Selected halogenated hydrocarbons. J. Chem. Eng. Data.

[30] Cibulka I., Takagi T. (2002). P−ρ−T data of liquids: Summarization and evaluation. 8. Miscellaneous compounds. J. Chem. Eng. Data.

[31] Reid R.C., Prausnitz J.M., Poling B.E., Company M.-H.B. (1987). The Properties of Gases and Liquids.

[32] Viswanath D.S., Ghosh T.K., Prasad D.H., Dutt N.V.K., Rani K.Y. (2007). Viscosity of Liquids: Theory, Estimation, Experiment, and Data.

[33] Lucas K. (1974). Ein einfaches verfahren zur berechnung der viskosität von Gasen und Gasgemischen. Chem. Ing. Tech..

[34] Assael M.J., Dymond J.H., Polimatidou S.K. (1994). Correlation and prediction of dense fluid transport coefficients. Fluid Phase Equilib..

[35] Cano-Gómez J.J., Iglesias-Silva G.A., Rico-Ramírez V., Ramos-Estrada M., Hall K.R. (2015). A new correlation for the prediction of refractive index and liquid densities of 1-alcohols. Fluid Phase Equilib..

[36] Pádua A.A.H., Fareleira J.M.N.A., Calado J.C.G., Wakeham W.A. (1996). Density and viscosity measurements of 2,2,4-trimethylpentane (isooctane) from 198 K to 348 K and up to 100 MPa. J. Chem. Eng. Data.

[37] Tyn M.T., Calus W.F. (1975). Estimating liquid molar volume. Processing.

[38] ChemSpider—Building Community for Chemists. http://www.chemspider.com.

[39] Korea Thermophysical Properties Data Bank (KDB). http://www.cheric.org/research/kdb/hcprop/cmpsrch.php.

[40] Design Institute for Physical Properties (DIPPR). http://dippr.byu.edu/.

[41] Yaws C.L. (2008). Thermophysical Properties of Chemicals and Hydrocarbons.

[42] LookChem.com—Look for Chemicals. http://www.lookchem.com.

[43] AspenTech (2007). Aspen Physical Property System—Physical Property Methods.

[44] Cordeiro J. (2015). Medição e Modelação de Difusividades em CO2 Supercrítico e Etanol.

[45] Joback K.G., Reid R.C. (1984). A Unified Approach to physical Property Estimation Using Multivariate Statistical Techniques.

[46] Joback K.G., Reid R.C. (1987). Estimation of pure-component properties from group-contributions. Chem. Eng. Commun..

[47] Somayajulu G.R. (1989). Estimation Procedures for Critical Constants. J. Chem. Eng. Data.

[48] Klincewicz K.M., Reid R.C. (1984). Estimation of critical properties with group contribution methods. AIChE J..

[49] Ambrose D. (1978). Correlation and estimation of vapour-liquid critical properties. I: Critical temperatures of organic compounds. NPL Technical Report Chem. 92.

[50] Ambrose D. (1979). Correlation and Estimation of Vapour-Liquid Critical Properties. II: Critical Pressure and Critical Volume. NPL Technical Report. Chem. 92.

[51] Green D.W., Perry R.H. (2008). Perry’s Chemical Engineers’ Handbook.

[52] Wen X., Qiang Y. (2001). A new group contribution method for estimating critical properties of organic compounds. Ind. Eng. Chem. Res..

[53] Valderrama J.O., Rojas R.E. (2009). Critical properties of ionic liquids. Revisited. Ind. Eng. Chem. Res..

[54] Lee B.I., Kesler M.G. (1975). A generalized thermodynamic correlation based on three-parameter corresponding states. AIChE J..

[55] Pitzer K.S., Lippmann D.Z., Curl R.F., Huggins C.M., Petersen D.E. (1955). The Volumetric and Thermodynamic Properties of Fluids. II. Compressibility Factor, Vapor Pressure and Entropy of Vaporization. J. Am. Chem. Soc..

[56] Zêzere B., Magalhães A.L., Portugal I., Silva C.M. (2018). Diffusion coefficients of eucalyptol at infinite dilution in compressed liquid ethanol and in supercritical CO_2_/ethanol mixtures. J. Supercrit. Fluids.

[57] Leite J., Magalhães A.L., Valente A.A., Silva C.M. (2018). Measurement and modelling of tracer diffusivities of gallic acid in liquid ethanol and in supercritical CO_2_ modified with ethanol. J. Supercrit. Fluids.

[58] Catchpole O.J., Von Kamp J.C. (1997). Phase equilibrium for the extraction of squalene from shark liver oil using supercritical carbon dioxide. Ind. Eng. Chem. Res..

[59] Liu H., Silva C.M., Macedo E.A. (1998). Unified approach to the self-diffusion coefficients of dense fluids over wide ranges of temperature and pressure-hard-sphere, square-well, Lennard-Jones and real substances. Chem. Eng. Sci..

[60] Cordeiro J., Magalhães A.L., Valente A.A., Silva C.M. (2016). Experimental and theoretical analysis of the diffusion behavior of chromium(III) acetylacetonate in supercritical CO_2_. J. Supercrit. Fluids.

[61] Burkov A. (2019). The Hundred-Page Machine Learning Book.

[62] Pedregosa F., Varoquaux G., Gramfort A., Michel V., Thirion B., Grisel O., Blondel M., Prettenhofer P., Weiss R., Dubourg V. (2011). Scikit-learn: Machine learning in Python. J. Mach. Learn. Res..

[63] Hastie T., Tibshirani R., Friedman J. (2009). The Elements of Statistical Learning.

[64] Altman N.S. (1992). An introduction to kernel and nearest-neighbor nonparametric regression. Am. Stat..

[65] Mitchell J.B.O. (2014). Machine learning methods in chemoinformatics. Wiley Interdiscip. Rev. Comput. Mol. Sci..

[66] Quinlan J.R. (1987). Simplifying decision trees. Int. J. Man. Mach. Stud..

[67] Müller A.C., Guido S. (2016). Introduction to Machine Learning with Python: A Guide for Data Scientists.

[68] Breiman L. (2001). Random forests. Mach. Learn..

[69] Friedman J.H. (2001). Greedy function approximation: A gradient boosting machine. Ann. Stat..

[70] Svetnik V., Wang T., Tong C., Liaw A., Sheridan R.P., Song Q. (2005). Boosting: An ensemble learning tool for compound classification and QSAR modeling. J. Chem. Inf. Model..

[71] Cooper E. (1992). Diffusion Coefficients at Infinite Dilution in Alcohol Solvents at Temperatures to 348 K and Pressures to 17 MPa.

[72] Pratt K.C., Wakeham W.A. (1975). The mutual diffusion coefficient for binary mixtures of water and the isomers of propanol. Proc. R. Soc. Lond. A.

[73] Sun C.K.J., Chen S.-H. (1987). Tracer diffusion in dense methanol and 2-propanol up to supercritical region: Understanding of solvent molecular association and development of an empirical correlation. Ind. Eng. Chem. Res..

[74] Man C.W. (2001). Limiting Mutual Diffusion of Nonassociated Aromatic Solutes.

[75] Tyn M.T., Calus W.F. (1975). Temperature and concentration dependence of mutual diffusion coefficients of some binary liquid systems. J. Chem. Eng. Data.

[76] Sarraute S., Gomes M.F.C., Pádua A.A.H. (2009). Diffusion coefficients of 1-alkyl-3-methylimidazolium ionic liquids in water, methanol, and acetonitrile at infinite dilution. J. Chem. Eng. Data.

[77] Hurle R.L., Woolf L.A. (1982). Tracer diffusion in methanol and acetonitrile under pressure. J. Chem. Soc. Faraday Trans..

[78] Wong C.-F., Hayduk W. (1990). Molecular diffusivities for propene in 1-butanol, chlorobenzene, ethylene glycol, and n-octane at elevated pressures. J. Chem. Eng. Data.

[79] Wong C.-F. (1989). Diffusion Coefficients of Dissolved Gases in Liquids.

[80] Kopner A., Hamm A., Ellert J., Feist R., Schneider G.M. (1987). Determination of binary diffusion coefficients in supercritical chlorotrifluoromethane and sulfurhexafluoride with supercritical fluid chromatography (SFC). Chem. Eng. Sci..

[81] Han P., Bartels D.M. (1996). Temperature dependence of oxygen diffusion in H2O and D2O. J. Phys. Chem..

[82] Tominaga T., Matsumoto S. (1990). Diffusion of polar and nonpolar molecules in water and ethanol. Bull. Chem. Soc. Jpn..

[83] Sun C.K.J., Chen S.H. (1986). Tracer diffusion in dense ethanol: A generalized correlation for nonpolar and hydrogen-bonded solvents. AIChE J..

[84] Suárez-Iglesias O., Medina I., Pizarro C., Bueno J.L. (2007). Diffusion of benzyl acetate, 2-phenylethyl acetate, 3-phenylpropyl acetate, and dibenzyl ether in mixtures of carbon dioxide and ethanol. Ind. Eng. Chem. Res..

[85] Lin I.-H., Tan C.-S. (2008). Diffusion of benzonitrile in CO_2_—Expanded ethanol. J. Chem. Eng. Data.

[86] Kong C.Y., Watanabe K., Funazukuri T. (2017). Measurement and correlation of the diffusion coefficients of chromium(III) acetylacetonate at infinite dilution in supercritical carbon dioxide and in liquid ethanol. J. Chem. Thermodyn..

[87] Zêzere B., Cordeiro J., Leite J., Magalhães A.L., Portugal I., Silva C.M. (2019). Diffusivities of metal acetylacetonates in liquid ethanol and comparison with the transport behavior in supercritical systems. J. Supercrit. Fluids.

[88] Funazukuri T., Yamasaki T., Taguchi M., Kong C.Y. (2015). Measurement of binary diffusion coefficient and solubility estimation for dyes in supercritical carbon dioxide by CIR method. Fluid Phase Equilib..

[89] Kong C.Y., Sugiura K., Natsume S., Sakabe J., Funazukuri T., Miyake K., Okajima I., Badhulika S., Sako T. (2020). Measurements and correlation of diffusion coefficients of ibuprofen in both liquid and supercritical fluids. J. Supercrit. Fluids.

[90] Snijder E.D., te Riele M.J.M., Versteeg G.F., van Swaaij W.P.M. (1995). Diffusion Coefficients of CO, CO_2_, N_2_O, and N_2_ in ethanol and toluene. J. Chem. Eng. Data.

[91] Kong C.Y., Watanabe K., Funazukuri T. (2013). Diffusion coefficients of phenylbutazone in supercritical CO_2_ and in ethanol. J. Chromatogr. A.

[92] Zêzere B., Iglésias J., Portugal I., Gomes J.R.B., Silva C.M. (2020). Diffusion of quercetin in compressed liquid ethyl acetate and ethanol. J. Mol. Liq..

[93] Pratt K.C., Wakeham W.A. (1974). The mutual diffusion coefficient of ethanol-water mixtures: Determination by a rapid, new method. Proc. R. Soc. Lond. A.

[94] Zêzere B., Silva J.M., Portugal I., Gomes J.R.B., Silva C.M. (2020). Measurement of astaxanthin and squalene diffusivities in compressed liquid ethyl acetate by Taylor-Aris dispersion method. Sep. Purif. Technol..

[95] Heintz A., Ludwig R., Schmidt E. (2011). Limiting diffusion coefficients of ionic liquids in water and methanol: A combined experimental and molecular dynamics study. Phys. Chem. Chem. Phys..

[96] Liu Q., Takemura F., Yabe A. (1996). Solubility and diffusivity of carbon monoxide in liquid methanol. J. Chem. Eng. Data.

[97] Lin I.-H., Tan C.-S. (2008). Measurement of diffusion coefficients of p-chloronitrobenzene in CO_2_-expanded methanol. J. Supercrit. Fluids.

[98] Funazukuri T., Sugihara T., Yui K., Ishii T., Taguchi M. (2016). Measurement of infinite dilution diffusion coefficients of vitamin K_3_ in CO_2_ expanded methanol. J. Supercrit. Fluids.

[99] Lee Y.E., Li F.Y. (1991). Binary diffusion coefficients of the methanol water system in the temperature range 30–40 °C. J. Chem. Eng. Data.

[100] Fan Y.Q., Qian R.Y., Shi M.R., Shi J. (1995). Infinite dilution diffusion coefficients of several aromatic hydrocarbons in octane and 2,2,4-trimethylpentane. J. Chem. Eng. Data.

[101] Sun C.K.J., Chen S.H. (1985). Diffusion of benzene, toluene, naphthalene, and phenanthrene in supercritical dense 2,3-dimethylbutane. AIChE J..

[102] Toriurmi M., Katooka R., Yui K., Funazukuri T., Kong C.Y., Kagei S. (2010). Measurements of binary diffusion coefficients for metal complexes in organic solvents by the Taylor dispersion method. Fluid Phase Equilib..

[103] Sun C.K.J., Chen S.H. (1985). Tracer diffusion of aromatic hydrocarbons in n-hexane up to the supercritical region. Chem. Eng. Sci..

[104] Funazukuri T., Nishimoton N., Wakao N. (1994). Binary diffusion coefficients of organic compounds in hexane, dodecane, and cyclohexane at 303.2-333.2 K and 16.0 MPa. J. Chem. Eng. Data.

[105] Chen S.H., Davis H.T., Evans D.F. (1981). Tracer diffusion in polyatomic liquids. II. J. Chem. Phys..

[106] Sun C.K.J., Chen S.H. (1985). Tracer diffusion of aromatic hydrocarbons in liquid cyclohexane up to its critical temperature. AIChE J..

[107] Chen B.H.C., Sun C.K.J., Chen S.H. (1985). Hard sphere treatment of binary diffusion in liquid at high dilution up to the critical temperature. J. Chem. Phys..

[108] Noel J.M., Erkey C., Bukur D.B., Akgerman A. (1994). Infinite dilution mutual diffusion coefficients of 1-octene and 1-tetradecene in near-critical ethane and propane. J. Chem. Eng. Data.

[109] Chen H.C., Chen S.H. (1985). Tracer diffusion of crown ethers in n-decane and n-tetradecane: An improved correlation for binary systems involving normal alkanes. Ind. Eng. Chem. Fundam..

[110] Chen S.H., Davis H.T., Evans D.F. (1982). Tracer diffusion in polyatomic liquids. III. J. Chem. Phys..

[111] Pollack G.L., Kennan R.P., Himm J.F., Stump D.R. (1990). Diffusion of xenon in liquid alkanes: Temperature dependence measurements with a new method. Stokes–Einstein and hard sphere theories. J. Chem. Phys..

[112] Matthews M.A., Rodden J.B., Akgerman A. (1987). High-temperature diffusion of hydrogen, carbon monoxide, and carbon dioxide in liquid n-heptane, n-dodecane, and n-hexadecane. J. Chem. Eng. Data.

[113] Matthews M.A., Akgerman A. (1987). Diffusion coefficients for binary alkane mixtures to 573 K and 3.5 MPa. AIChE J..

[114] Rodden J.B., Erkey C., Akgerman A. (1988). High-temperature diffusion, viscosity, and density measurements in n-eicosane. J. Chem. Eng. Data.

[115] Qian R.Y., Fan Y.Q., Shi M.R., Shi J. (1996). Predictive equation of tracer liquid diffusion coefficient from viscosity. Chin. J. Chem. Eng..

[116] Li S.F.Y., Wakeham W.A. (1989). Mutual diffusion coefficients for two n-octane isomers in n-heptane. Int. J. Thermophys..

[117] Grushka E., Kikta E.J. (1976). Diffusion in liquids. II. Dependence of diffusion coefficients on molecular weight and on temperature. J. Am. Chem. Soc..

[118] Lo H.Y. (1974). Diffusion coefficients in binary liquid n-alkane systems. J. Chem. Eng. Data.

[119] Alizadeh A.A., Wakeham W.A. (1982). Mutual diffusion coefficients for binary mixtures of normal alkanes. Int. J. Thermophys..

[120] Padrel de Oliveira C.M., Fareleira J.M.N.A., Nieto de Castro C.A. (1989). Mutual diffusivity in binary mixtures of n-heptane with n-hexane isomers. Int. J. Thermophys..

[121] Li S.F.Y., Yue L.S. (1990). Composition dependence of binary diffusion coefficients in alkane mixtures. Int. J. Thermophys..

[122] Matthews M.A., Rodden J.B., Akgerman A. (1987). High-temperature diffusion, viscosity, and density measurements in n-hexadecane. J. Chem. Eng. Data.

[123] Awan M.A., Dymond J.H. (1996). Transport properties of nonelectrolyte liquid mixtures. X. Limiting mutual diffusion coefficients of fluorinated benzenes in n-hexane. Int. J. Thermophys..

[124] Okamoto M. (2002). Diffusion coefficients estimated by dynamic fluorescence quenching at high pressure: Pyrene, 9,10-dimethylanthracene, and oxygen in n-hexane. Int. J. Thermophys..

[125] Dymond J.H., Woolf L.A. (1982). Tracer diffusion of organic solutes in n-hexane at pressures up to 400 MPa. J. Chem. Soc. Faraday Trans. 1.

[126] Safi A., Nicolas C., Neau E., Chevalier J.L. (2007). Measurement and correlation of diffusion coefficients of aromatic compounds at infinite dilution in alkane and cycloalkane solvents. J. Chem. Eng. Data.

[127] Leffler J., Cullinan H.T. (1970). Variation of liquid diffusion coefficients with composition. Dilute ternary systems. Ind. Eng. Chem. Fundam..

[128] Harris K.R., Pua C.K.N., Dunlop P.J. (1970). Mutual and tracer diffusion coefficients and frictional coefficients for systems benzene-chlorobenzene, benzene-n-hexane, and benzene-n-heptane at 25 °C. J. Phys. Chem..

[129] Bidlack D.L., Kett T.K., Kelly C.M., Anderson D.K. (1969). Diffusion in the solvents hexane and carbon tetrachloride. J. Chem. Eng. Data.

[130] Grushka E., Kikta E.J. (1974). Extension of the chromatographic broadening method of measuring diffusion coefficients to liquid systems. I. Diffusion coefficients of some alkylbenzenes in chloroform. J. Phys. Chem..

[131] Holmes J.T., Olander D.R., Wilke C.R. (1962). Diffusion in mixed Solvents. AIChE J..

[132] Funazukuri T., Ishiwata Y. (1999). Diffusion coefficients of linoleic acid methyl ester, Vitamin K3 and indole in mixtures of carbon dioxide and n-hexane at 313.2 K, and 16.0 MPa and 25.0 MPa. Fluid Phase Equilib..

[133] Moore J.W., Wellek R.M. (1974). Diffusion coefficients of n-heptane and n-decane in n-alkanes and n-alcohols at several temperatures. J. Chem. Eng. Data.

[134] Márquez N., Kreutzer M.T., Makkee M., Moulijn J.A. (2008). Infinite dilution binary diffusion coefficients of hydrotreating compounds in tetradecane in the temperature range from (310 to 475) K. J. Chem. Eng. Data.

[135] Debenedetti P.G., Reid R.C. (1986). Diffusion and mass transfer in supercritical fluids. AIChE J..

