# Supplementary material for: Predictive Models for the Binary Diffusion Coefficient at Infinite Dilution in Polar and Nonpolar Fluids
Source: Materials (Basel). 2021 Jan 23;14(3):542. doi: 10.3390/ma14030542 (PMC7866074; doi:10.3390/ma14030542)
Supplement: Supplementary file 1 [file materials-14-00542-s001.pdf]

materials

MDPI

Supplementary Material

Predictive Models for the Binary Diffusion Coefficient at Infinite Dilution in Polar and Nonpolar Fluids

José P. S. Aniceto, Bruno Zêzere and Carlos M. Silva *

CICECO—Aveiro Institute of Materials, Department of Chemistry, University of Aveiro, 3810-193 Aveiro, Portugal; joseaniceto@ua.pt (J.P.S.A.); brunozezere@ua.pt (B.Z.)
* Correspondence: carlos.manuel@ua.pt

Table of Contents

Software........ 1
Table S1........ 2
Figure S1........ 2
Figure S2........ 3
Figure S3........ 3
Figure S4........ 4
Figure S5........ 4
Figure S6........ 5
Figure S7........ 5
Figure S8........ 6
Figure S9........ 6
Figure S10........ 7
Figure S11........ 7
Figure S12........ 8

Citation: Aniceto, J.P.S.; Zêzere, B.; Silva, C.M. Predictive Models for the Binary Diffusion Coefficient at Infinite Dilution in Polar and Nonpolar Fluids. *Materials* **2021**, *14*, 542. https://doi.org/10.3390/ma14030542

Received: 23 December 2020
Accepted: 19 January 2021
Published: 23 January 2021

Publisher's Note: MDPI stays neutral with regard to jurisdictional claims in published maps and institutional affiliations.

Copyright: © 2021 by the authors. Submitted for possible open access publication under the terms and conditions of the Creative Commons Attribution (CC BY) license (http://creativecommons.org/licenses/by/4.0/).

Software

The two models developed in this work (for polar and nonpolar systems) are provided as a Python command line interface tool. Download and usage instructions can be found in either of the following links:

- https://www.egichem.com/tools/calculators/d12-polar-nonpolar/
- https://github.com/EgiChem/ml-D12-app

Table S1. Tested and best hyper-parameter values for each machine learning algorithm.

ML Algorithm	Hyper-parameter	Values Tested	Best
k-Nearest Neighbors	Number of neighbors	3–15	3
	Algorithm	auto; ball_tree; kd_tree; brute	auto
	Leaf size (BallTree or KDTree algorithm)	3; 5; 15; 30; 40; 50	15
	Weight function	uniform; distance	distance
Decision Tree	Quality of a split metric	mse; mae; friedman_mse	mae
	Split strategy	best; random	best
	Maximum depth of the tree	None; 2; 8; 12	None
	Minimum number of samples per leaf	0.1; 2; 5	1
	Minimum number of samples to split a node	0.1; 2; 8	2
	Minimum weighted fraction required for leaf node	0; 0.1; 0.5	0
	Maximum number of features for split	auto; sqrt; log2; None	auto
	Minimum impurity decrease	0; 0.5; 2	0
Random Forest	Quality of a split metric	mse; mae	mse
	Number of estimators	10; 15; 20; 30; 100; 150	20
	Maximum depth of the tree	None; 2; 10	None
	Minimum number of samples per leaf	0.1; 1; 2; 5	1
	Minimum number of samples to split a node	0.1; 2; 4	2
	Minimum weighted fraction required for leaf node	0; 0.1; 0.5	0
	Maximum number of features for best split	auto; sqrt; log2; None	log2
	Minimum impurity decrease	0; 0.5; 2	0
	Bootstrap samples when building trees	True; False	False
Gradient Boosted	Quality of a split metric	friedman_mse; mse; mae	mae
	Loss function	ls; lad; huber	ls
	Learning rate	0.01; 0.1; 0.5	0.01
	Number of trees used in the boosting process	100, 500, 1500, 2500	2500
	Maximum depth of each tree	2; 3; 8; 10; 12	10
	Minimum number of samples per leaf	1; 2; 4	1
	Minimum number of samples to split a node	2; 6; 10; 16	16
	Maximum number of features for split	auto; sqrt; log2; None	log2

mse: mean squared error; mae: mean absolute error; ls: least square regression; lad: least absolute deviation; huber: a combination of ls and lad.

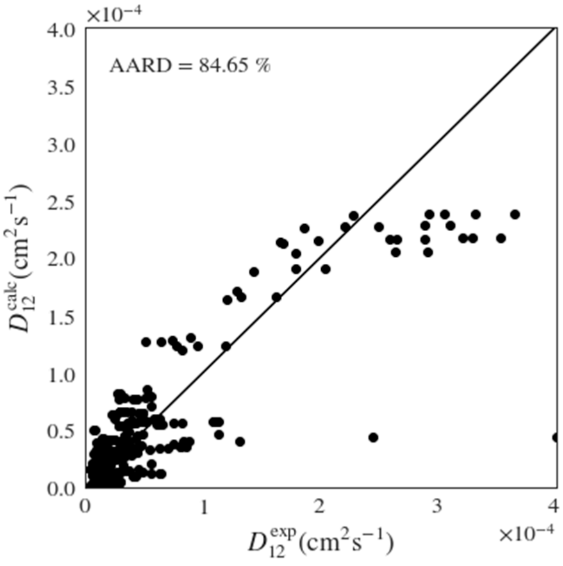

Figure S1. Predicted *versus* experimental diffusivities for the test set of polar systems using the Multilinear Regression model.

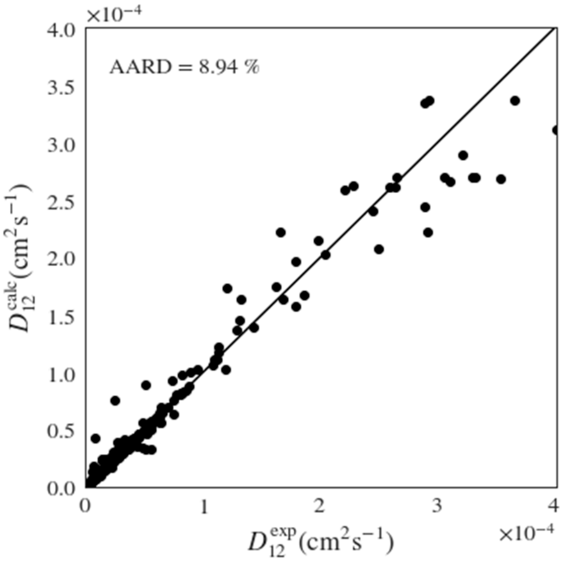

Figure S2. Predicted *versus* experimental diffusivities for the test set of polar systems using the *k*-Nearest Neighbors model.

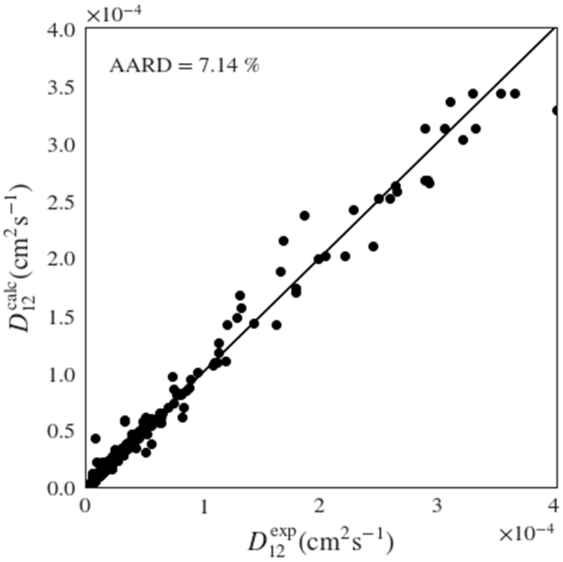

Figure S3. Predicted *versus* experimental diffusivities for the test set of polar systems using the Decision Tree model.

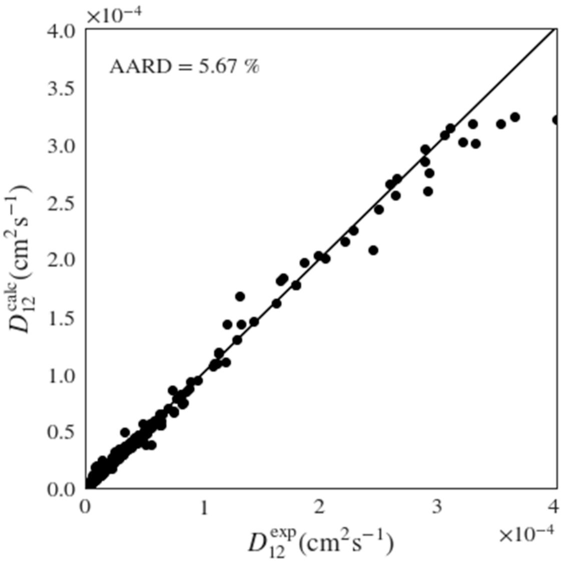

Figure S4. Predicted *versus* experimental diffusivities for the test set of polar systems using the Random Forest model.

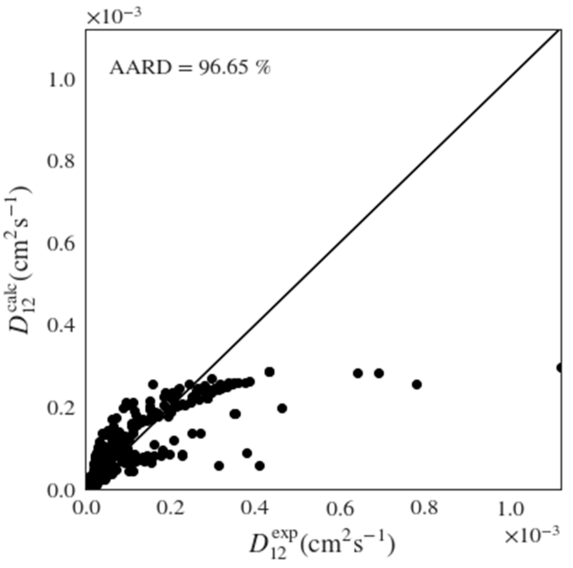

Figure S5. Predicted versus experimental diffusivities for the test set of nonpolar systems using the Multilinear Regression model.

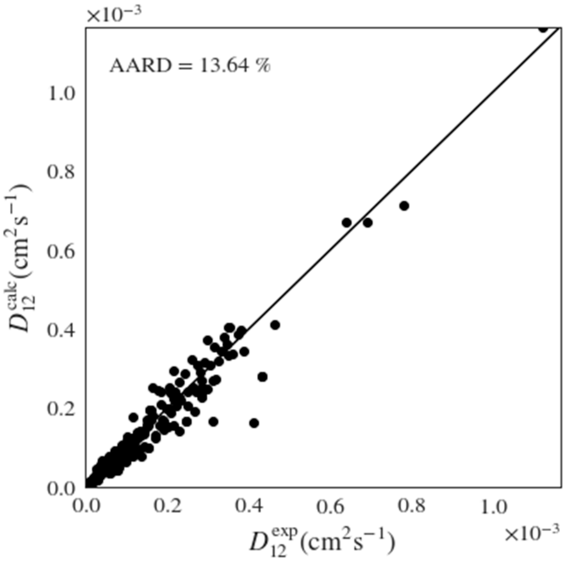

Figure S6. Predicted *versus* experimental diffusivities for the test set of nonpolar systems using the *k*-Nearest Neighbors model.

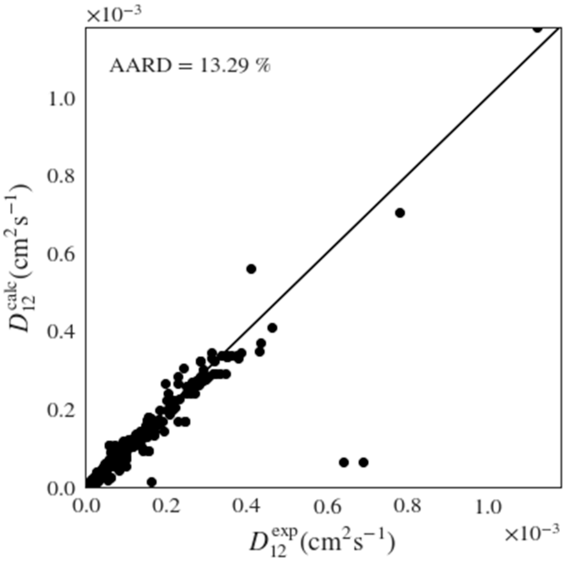

Figure S7. Predicted *versus* experimental diffusivities for the test set of nonpolar systems using the Decision Tree model.

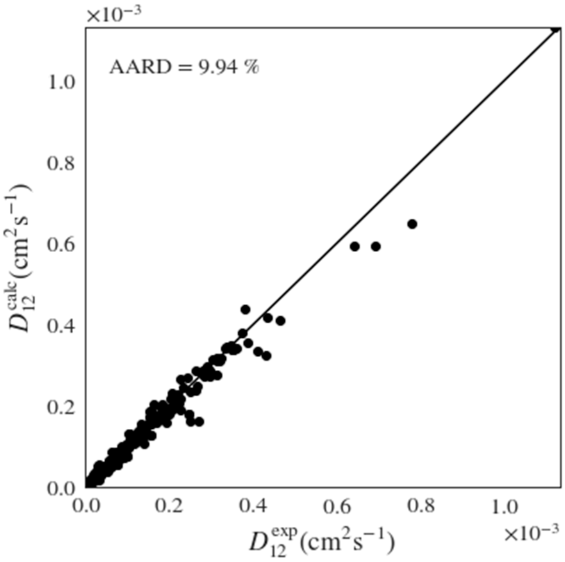

Figure S8. Predicted *versus* experimental diffusivities for the test set of nonpolar systems using the Random Forest model.

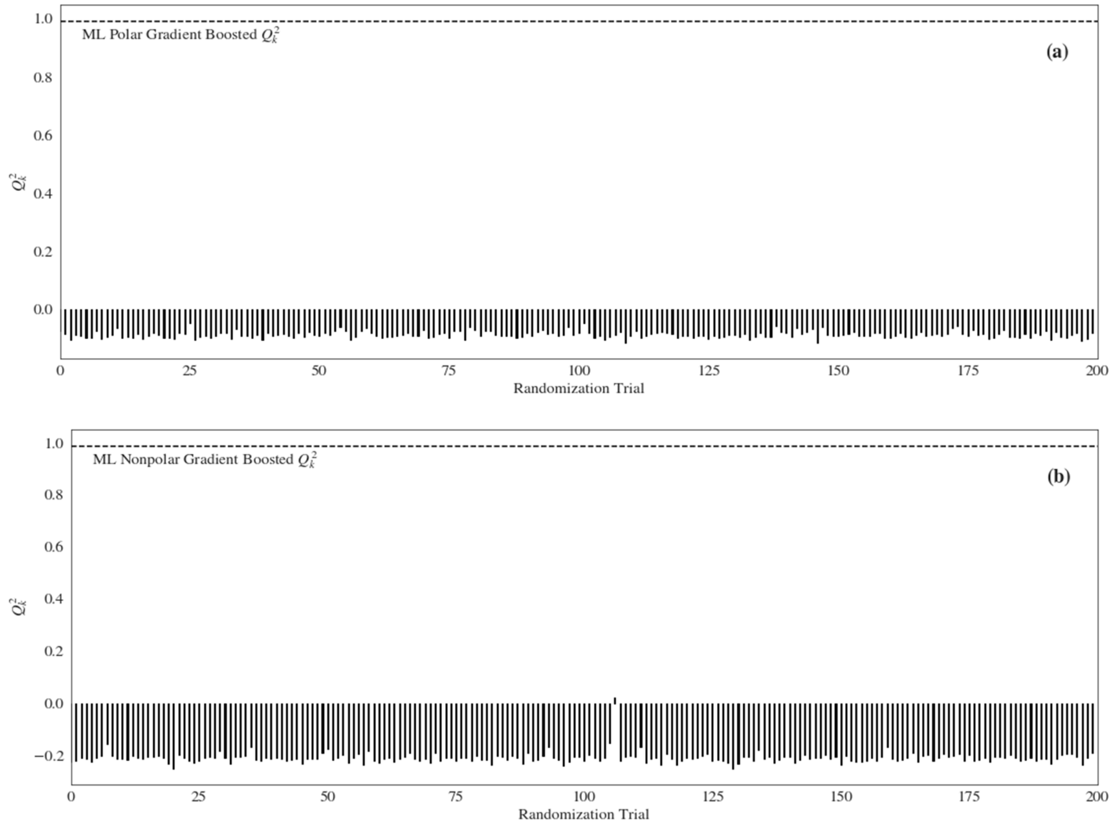

Figure S9. y-Randomization calculations for the selected ML Gradient Boosted models for (**a**) polar systems and (**b**) non-polar systems. The bars show the Q^2 values for optimized models based on randomized diffusivity data. The dashed horizontal lines show the Q^2 values of the actual models.

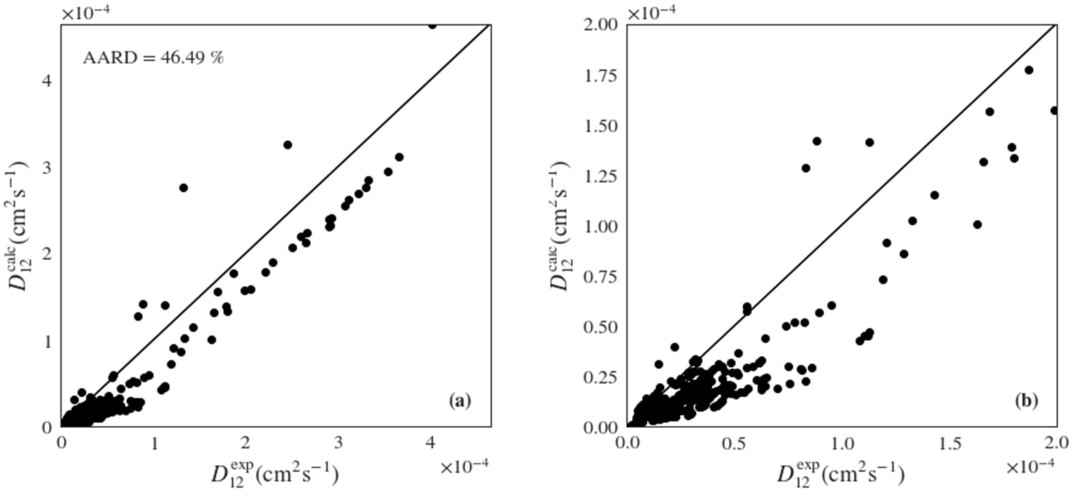

Figure S10. Calculated *versus* experimental diffusivities for the test set of polar systems for the Tyn-Calus model. (**a**) full D_{12} range; (**b**) zoomed on lower D_{12} range.

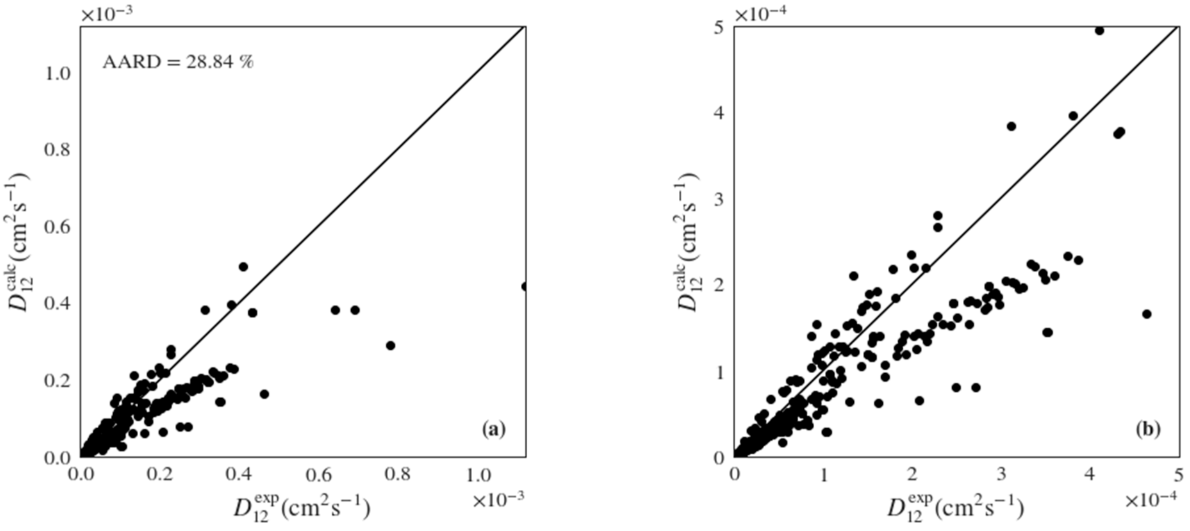

Figure S11. Calculated *versus* experimental diffusivities for the test set of nonpolar systems for the Tyn-Calus model. (**a**) full D_{12} range; (**b**) zoomed on lower D_{12} range.

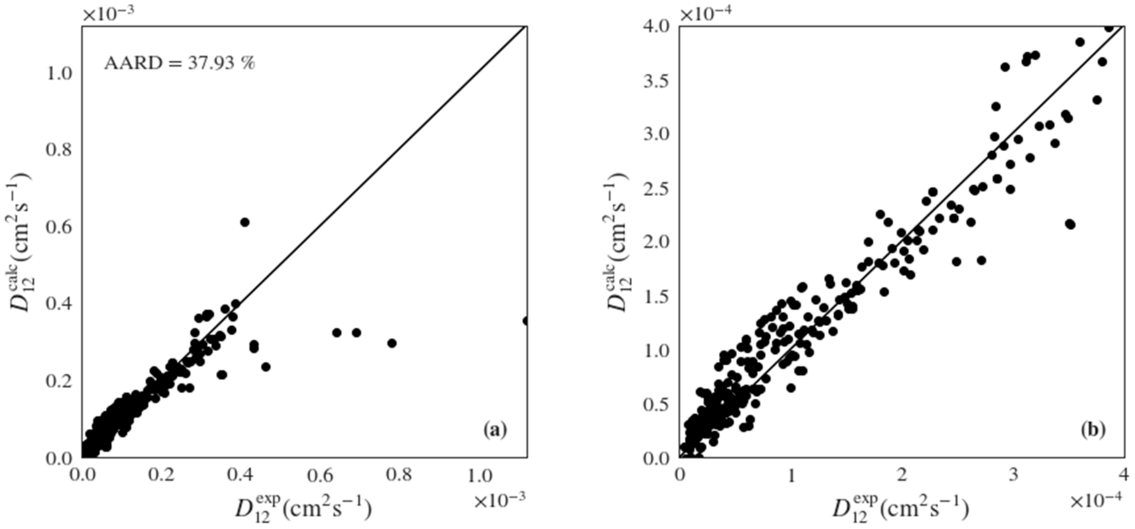

Figure S12. Calculated *versus* experimental diffusivities for the test set of nonpolar systems for the Zhu *et al.* model. (**a**) full D_{12} range; (**b**) zoomed on lower D_{12} range.
